# Exploring Relationships: A Systematic Review on Intimate Partner Violence and Attachment

**DOI:** 10.3389/fpsyg.2018.01166

**Published:** 2018-07-05

**Authors:** Patrizia Velotti, Sara Beomonte Zobel, Guyonne Rogier, Renata Tambelli

**Affiliations:** ^1^Department of Educational Sciences, Università di Genova, Genoa, Italy; ^2^Department of Dynamic and Clinical Psychology, Sapienza Università di Roma, Rome, Italy

**Keywords:** attachment, intimate partner violence, systematic review, victimization, perpetration, mutual violence, homosexuality

## Abstract

**Background:** Intimate Partner Violence (IPV) is an important public health challenge. In recent years, there has been a greater awareness concerning this phenomenon, its causes and consequences. Due to the relational nature of IPV, attachment theory (Bowlby, 1988) appears a useful framework to read the phenomenon and to better understand its components and its dynamics to provide more precise and tailored interventions in the future.

**Purpose:** To summarize our knowledge of the research concerning IPV and attachment with an aim to better design and implement future research.

**Methods:** Computer database researches were conducted using the following databases: Psychinfo, Psycharticle, Medline, Scopus, Web of Science, and PubMed (all years to the 01/02/2018). Search terms were compiled into two concepts for all database namely Attachment and IPV.

**Results:** After removing the duplicates, a total of 3,598 records was screened, resulting in the identification of 319 full-text articles to be further scrutinized. Upon closer examination, there was consensus that 113 of those studies met the study inclusion criteria. Data was organized considering specifically studies concerning (1) IPV victimization and attachment, (2) IPV perpetration and attachment (both these sections were articulated in Physical, Psychological, and Sexual IPV), and (3) New research (comprising same-sex couples, IPV and attachment in couple contexts and IPV profiles and attachment among perpetrators).

**Conclusion:** A number of studies failed to find significant associations between insecure attachment and IPV victimization or perpetration. Additional research is needed to provide a greater understanding of different IPV forms and to aid in the development of prevention and treatment interventions.

## Introduction

### Rationale

Intimate Partner Violence (IPV) is defined by World Health Organization (WHO) as “any behavior within an intimate relationship that cause physical, physical or sexual harm to those in the relationship” (Heise et al., 1999). The term IPV comprises different forms of violence, that go from manipulation to sexual coercion, that can be divided in three main categories: physical violence, psychological violence and sexual violence.

To understand violence, due to its complexity, the ecological model was applied: IPV seems to be a result of the explosive interaction between individual, relational, community and societal factors (Garcia-Moreno et al., 2005).

Physical and mental health are affected by IPV through both direct pathways, like wounds and injuries, and indirect pathways, like chronic health problems or psychological consequences of trauma and stress (Krug et al., 2002).

Due to the relational nature of IPV, we thought that Attachment Theory can be a useful framework to read the phenomenon and to better understand its components and its dynamics to provide more precise and tailored interventions in the future. At the end of the Eighties, attachment theory has also been used to investigate the quality of adult attachment relationships (Hazan and Shaver, 1987; Mikulincer and Shaver, 2007). Individual differences in adult attachment are assessed via self-report (e.g., Brennan et al., 1998) or interview (e.g., George et al., 1985; Velotti et al., 2011; Castellano et al., 2014). In both these traditions individuals can be classified into categories—secure, insecure-dismissing, insecure-preoccupied, disorganized—corresponding to those obtained among children. Also, research suggests that adult attachment is best described by two dimensions, avoidance, and anxiety (Shaver and Mikulincer, 2002; Mikulincer and Shaver, 2007). Individuals scoring high on the avoidance dimension are characterized by feelings of fear and uneasiness regarding intimacy as well as the difficulty to accept dependency on others within an affective bond (for example discomfort when the partner becomes too intimate or dependent). High scores on the anxiety dimension appear to reflect preoccupation about the reliability of the attachment figure and the availability to face the needs of attachment (for example, one might think the partner may be interested in someone else or that he/she does not desire closeness). The combination of anxiety and avoidance leads to four prototypes (Brennan et al., 1998): the secure (low levels of avoidance and low levels of anxiety), preoccupied (low levels of avoidance, but high levels of anxiety), dismissing (which is the same as the avoidant style mentioned above, with high levels of avoidance and low levels of anxiety), and lastly, the fearful style.

Each one of these lines of research has contributed to enrich the knowledge of the mechanisms, which come into play in the formation, functioning, and evolution of couple relationships (Mikulincer and Shaver, 2007).

Concerning IPV in romantic relationships, violence has been interpreted by researchers as a dysfunctional attempt to maintain proximity to the partner, that assumes the role of an attachment figure, when attachment needs are threatened (Simpson and Rholes, 1994).

According to Shaver and Mikulincer (2011), people with anxious attachment would tend to be ambivalent toward power and domination; on one hand, in fact, they would like to have control of the relationship, but on the other they may fear to obtain it, because this could provoke the resentment of the partner, and therefore constitute a threat to the stability of the relationship. People with an avoidant attachment would instead tend toward autonomy and distance, the critical vision of others, and the perception of others as objects to be used instrumentally for the satisfaction of their needs.

The joint between insecurely attached partners is peculiar: one partner may perceive a threat when the other partner claims for autonomy, as if leaving he won't ever get back again, and he gains reassurance only maintaining proximity and control over him. In reverse, the other partner may perceive partner's need for closeness and intimacy as oppressing and threatening for its autonomy. This conflicting perspectives can easily lead to a misunderstanding that often generates violence, perpetrated by one partner or both (Hazan and Shaver, 1987).

### Objectives

In the years, several reviews of different nature have been conducted to explore the relationship between the two constructs.

McClellan and Killeen (2000) produced the first narrative review exploring the use of aggression by males in couples in the light of attachment theory: the paper is focused on the evidence that adults internal working models have a consistent role in their adult relationship with intimate partners, making a parallel between infant experiences of attachment and the replications of insecure patterns in adulthood.

A review on risk and protective factors for male psychological abuse toward partners has been conducted by Schumacher et al. (2001): according to this review, adult attachment, along with other factors such as communication partners and marital adjustment, is significantly associated with psychological IPV.

A review on literature on female perpetrators of IPV has been written by Carney et al. (2007): the narrative review makes an interesting confrontation on male and female offenders and includes a summary of existing intervention programs for these women.

Finkel and Slotter (2006) have discussed a narrative review, adopting an attachment perspective to reconsider IPV as an impulsive behavior that occurs when an individual feels threatened in the relationship.

Langhinrichsen-Rohling (2010) wrote an interesting paper on controversial discussions regarding gender and IPV in US, addressing topics about subtypes of IPV, differences between male and female perpetrators and gender-related challenges concerning the phenomenon.

Ogilvie et al. (2014) wrote a meta-analysis focused on attachment and violent offending, investigating controversial results about the correlation between attachment and several typologies of criminal offending (i.e., IPV, violent offending, sexual offending, non-violent offending).

The narrative review produced by Park (2016) is a very useful dissertation about implications of attachment theory applications to IPV, focused on theory's strengths and limitation in both understanding and facing the phenomenon.

Tapp and Moore (2016) produced a very useful article on instruments to assess the risk of IPV in late adolescents and young adults. It provides a very exhaustive review on the most used measures, highlighting their characteristics and efficacy, to explore the phenomenon and detect risk potential.

In the end, Karantzas et al. (2016) provided a very complete systematic review concerning the topic of attachment style and less severe forms of sexual coercion, taking in consideration the phenomenon not only in couple setting but also related to acts perpetrated toward other people.

After exploring existing reviews discussing the relationship between IPV and attachment, we found a gap in research: no other review examined studies that explored the relationship between attachment and IPV in all its manifestations nor it adopted a systematic approach nor it considered studies conducted among male and female samples.

Therefore, this systematic review has the objective to collect and draw conclusions from all the studies available that investigated the relationship between attachment and all forms of IPV, considering researches conducted among male and female samples and not only among couples. It is crucial for both clinicians and researchers to have a clearer view of the correlations between the two constructs, with the goal to elaborate specific programs, to prevent and to intervene properly on both perpetrators and victims.

### Research question

Two main research questions have led to the preparation of this review: Is attachment involved in IPV? How does attachment explain the process that leads to IPV?

## Method

**Figure 1:**
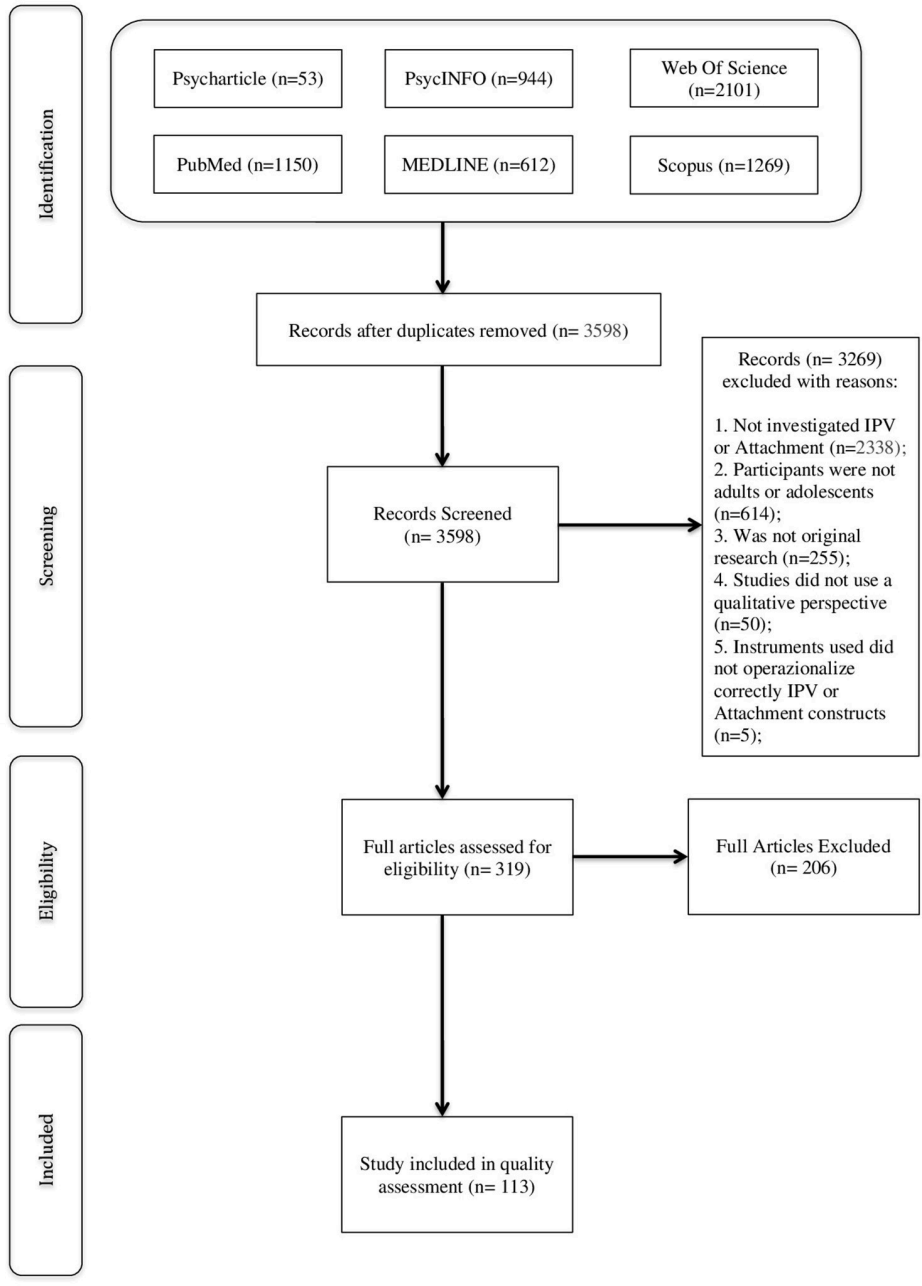
Flow diagram describing the processes of identification, screening and inclusion of the studies.

A systematic search was conducted according to PRISMA guidelines (Moher et al., 2009). The full process of study identification, inclusion and exclusion is illustrated in Figure 1.

### Search strategy

Computer database researches were conducted using the following databases: Psychinfo, Psycharticle, Medline, Scopus, Web of Science, and PubMed (all years to the 01/02/2018). Search terms were compiled into two concepts for all database namely Attachment and IPV. For Psycinfo, Psycharticle, Medline and Scopus, terms of Appendix A were searched in Title, Abstract and Key-words fields and results were refined including only articles. For Web of Science, terms presented in Appendix B were searched into Topic field. Then, we refined results including articles and excluding the Medline database. Finally, for PubMed, terms presented in Appendix B (including Mesh Terms) were searched into Title and Abstract fields.

### Selection of studies

We screened every title and abstract to determine the eligibility of the study for inclusion. Criteria for inclusion of studies were the following: (1) To investigate both attachment and IPV constructs; (2) To conduct study on adults or adolescents; (3) To provide original research; (4) To use a qualitative perspective; (5) To use validated instruments for the measurement of both attachment and IPV.

Two reviewers (SBZ, GR) independently conducted the electronic searches using the aforementioned databases. Together, independent review of these electronic databases identified a total of 6,129 articles with the initial search terms, which were then examined by each reviewer for eligibility. After removing the duplicates, a total of 3,598 records was screened, resulting in the identification of 319 full-text articles to be further scrutinized. Upon closer examination, there was consensus that 113 of those studies met the study inclusion criteria.

### Data extraction and reporting

A coding protocol was prepared and used to extract relevant information from the selected primary studies. In particular, six classes of information were coded: (1) characteristics of the publication (i.e., year); (2) characteristics of the sample (i.e., total sample size; gender; age was coded as the mean, standard deviation in years, sample composition); (3) information about the methodological characteristics (i.e., the context of the study was coded as the country in which the research was conducted; the type of design was coded as cross-sectional or longitudinal; the instruments used to measure attachment and IPV were reported) (4) Main results (the dimensions of attachment significantly associated with IPV were reported together with the statistical index used in the study).

## Results

### IPV victimization and attachment

We found 47 studies examining attachment among victims of IPV. Despite the fact that the first papers on the topic were written on 1997, 72.92% of the studies have been published in the last 10 years. Importantly, 1.46% of these studies did not distinguish between different forms of IPV. In contrast, 60.42% of the papers focused on physical IPV, 45.83% examined psychological forms of violence and only 1.25% investigated sexual IPV. Results are presented within these four categories in the following sections.

#### Generic IPV victimization and attachment

Table 1 displays the characteristics of the seven studies investigating attachment among victims of IPV without distinguishing between the different forms of violence. Most of researches have been conducted in USA (57.14%) and Canada (42.86%). Research is mainly cross-sectional with only two contributions adopting a longitudinal prospective (Weiss et al., 2011; La Flair et al., 2015).

**Table 1:** Studies investigating the relationship between generic IPV and attachment among victims.

**References**	**Country**	**Design**	**Sample characteristics**				**Instrument used to evaluate IPV**	**Instrument used to evaluate attachment**	**Main results**
			**Gender composition**	**Size**	**Type**	**Age**			
Wekerle and Wolfe, 1998	Canada	Cross-sectional	Female	193	High school students	15.13 (0.94)	CIRQ	ASR	Avoidance (*r* = 0.28) Anxiety (*r* = 0.19)
			Male	128		15.34 (1.75)			
Frey et al., 2011	USA	Cross-sectional	Female	20	Couples with men in military service	28.50 (5.11)	IJS	MIMARA	Avoidance (*r* = 0.32 for F) Anxiety (*r* = −0.03 for F)
			Male	20		28.20 (6.24)			
Weiss et al., 2011	Canada	Longitudinal	Female	90	With intellectual disability	15.8 (0.98)	CADRI	ASR	Security (*r* = −0.20) Avoidance (*r* = 0.33)
			Male	66					
Shechory, 2013	Israel	Cross-sectional	Female	36	With history of IPV	35.65 (8.53)	3 questions	ECR	Anxiety (*F* = 14.83; *p* < 0.05) Avoidance (*F* = 10.26; *p* < 0.05)
			Female	89	Without history of IPV				
Yarkovsky and Timmons Fritz, 2014	Canada	Cross-sectional	Female	137	Undergraduate students	20.76 (1.87)	CADRI	ECR	Anxiety (*r* = 0.30)
La Flair et al., 2015	USA	Longitudinal	Female	215	Healthcare workers	39.7 (11.26)	AAS	ECR-R	Anxiety (*r* = 0.29) Avoidance (*r* = 0.27)
Lewis et al., 2017	USA	Cross-sectional	Female	293	Couples with pregnant F with and without story of IPV	18.7 (1.7)	CTS2	ECR	Anxiety (*F* = 5.66; *p* < 0.05)
			Male	293		21.3 (4.1)			
McClure and Parmenter, 2017	USA	Cross-sectional	Female	161	Undergraduate students	18.83 (1.03)	CADRI	AAS (only Anxiety scale)	Anxiety (*r* = 0.13)
			Male	93					

*IPV, Intimate Partner Violence; CIRQ, Conflict in Relationships Questionnaire; ASR, Attachment Security Ratings; IJS, Intimate Justice Scale; MIMARA, Multi Measure of Adult Romantic Attachment; F, Female; M, Male; CADRI, Conflict in Adolescent Dating Relationships Inventory; ECR, Experiences in Close Relationship Questionnaire; AAS, Abuse Assessment Screen; ECR-R, Experiences in Close Relationship Questionnaire Revised; CTS2, Conflict Tactics Scale Revised; AAS, Adult Attachment Scale*.

Researchers generally decided to investigate the topic among samples balanced for gender with only two exceptions (Shechory, 2013; Yarkovsky and Timmons Fritz, 2014). Also, they often used participants with no previous report of IPV such as students (Wekerle and Wolfe, 1998; Yarkovsky and Timmons Fritz, 2014; McClure and Parmenter, 2017) or participants recruited among minority populations. For example, some studies have been conducted on couples with men doing military service (Frey et al., 2011), on adolescents with intellectual disability (Weiss et al., 2011) and on healthcare female workers (La Flair et al., 2015). Noteworthy, only two studies recruited participants with a previous reported history of IPV (Shechory, 2013; Lewis et al., 2017).

Also, studies showed heterogeneity regarding age of the participants with three studies investigating the topic among adult population (Frey et al., 2011; Shechory, 2013; La Flair et al., 2015), and five studies recruiting young adults or adolescents (Wekerle and Wolfe, 1998; Weiss et al., 2011; Yarkovsky and Timmons Fritz, 2014; Lewis et al., 2017; McClure and Parmenter, 2017).

Studies were highly heterogeneous regarding the instrument used to measure IPV with the most administering the Conflict in Adolescents Dating Relationships (CADRI, Wolfe et al., 2001). In contrast, measurment of attachment appeared more consistent with the Experiences in Close Relationships (ECR; Brennan et al., 1998) and its revised version being the most used.

Results seem to support the hypothesis of a relationship between the attachment dimensions anxiety and avoidance and IPV victimization. Indeed, almost all correlational studies, with two exceptions (Frey et al., 2011; Weiss et al., 2011), found significant and positive correlation between the anxious dimension of attachment and IPV victimization. However, coefficient indicated only weak associations, ranging from 0.13 to 0.30. The fact that Weiss et al. (2011) did not replicate this result may be explained by the specificity of their sample, being constituted by adolescents with intellectual disability. Also, Frey et al. (2011) found a negative and significant correlation (albeit very weak: *r* = −0.03) between IPV victimization and anxious attachment in female partners of men doing military service. It has to be noted that the sample in this study is particularly small and results are consequently difficult to generalize to the whole population. Supporting the idea that victims of IPV may have high levels of anxious attachment, two studies successfully compared groups of females with a history of IPV with groups of females without previous reported victimization (Shechory, 2013; Lewis et al., 2017). Both found that females belonging to the IPV group scored higher on the anxious dimension of attachment compared to control participants.

Turning to the attachment dimension of avoidance, results are more contrasting with five studies showing associations with IPV victimization (Wekerle and Wolfe, 1998; Frey et al., 2011; Weiss et al., 2011; Shechory, 2013; La Flair et al., 2015) and others failing to replicate such results (Yarkovsky and Timmons Fritz, 2014; Lewis et al., 2017; McClure and Parmenter, 2017). As for the anxiety dimension, correlational coefficients indicate weak associations between avoidance and IPV victimization, ranging from 0.27 to 0.33.

Moreover, some of these studies brought additional contributions for the understanding of the relationship between IPV and attachment. For example, the role of individual differences has been pointed out, underlying that intellectual ability (Weiss et al., 2011) and gender (Wekerle and Wolfe, 1998; Lewis et al., 2017) may play a moderating role in such link. Also, two studies, using structural equation modeling, evidenced that attachment insecurity may mediate the relationship between IPV and psychological symptoms as Post-Traumatic Stress Disorder (PTSD) (Frey et al., 2011) or depression (La Flair et al., 2015). Importantly, a study showed that attachment insecurity predicted no longer IPV victimization after controlling for social desirability (Yarkovsky and Timmons Fritz, 2014).

#### Physical IPV victimization and attachment

Studies examining attachment in victims of physical IPV were 30 (all displayed in Table 2) with 62% conducted in USA, 24.14% in Europe, 10.34% in Canada and one in Chile. Only five studies adopted a longitudinal design of research with the others being cross-sectional.

**Table 2:** Studies investigating the relationship between physical IPV and attachment among victims.

**References**	**Country**	**Design**	**Sample characteristics**				**Instrument used to evaluate IPV**	**Instrument used to evaluate attachment**	**Attachment's dimensions resulting associated with IPV**
			**Gender composition**	**Size**	**Type**	**Age**			
Henderson et al., 1997	Canada	Longitudinal	Female	63	With history of IPV	31.4 (NA)	CTS	Interview coded with the Bartholomew's model	Fearful (*r* = 0.23)
Bookwala, 2002	USA	Cross-sectional	Female	102	Undergraduate students	NA	CTS2	RQ	NSO
			Male	59					
Bond and Bond, 2004	Canada	Cross-sectional	Female	43	Couples	39.85 (10.26)	MSI-R PAPS	RQ ECR	Anxiety (*r* = 0.42 for F; *r* = −0.32 for M) Avoidance (*r* = 0.38 for M)
			Male	43		41.83 (11.48)			
Henderson et al., 2005	Canada	Cross-sectional	Male and Female	128	Community participants	37.4 (12.6)	CTS2	HAI	Secure (*r* = −0.18) Preoccupied (*r* = 0.23)
Orcutt et al., 2005	USA	Cross-sectional	Female	457	College students	NA	CTS2	ECR-R	NSO
Rogers et al., 2005	USA	Cross-sectional	Female	80	Couples College students	19.54 (3.40)	HAQ	AAQ	Avoidance (Statistical index NA)
			Male	80		20.71 (3.66)			
Toews et al., 2005	USA	Cross-sectional	Female	147	Divorced mothers	34 (NA)	CTS2	RSQ	Insecurity (*r* = 0.30)
Shurman and Rodriguez, 2006	USA	Cross-sectional	Female	85	Help-seeking victims of IPV	33.89 (9.6)	ABI	ASQ	NSO
Higginbotham et al., 2007	USA	Cross-sectional	Female	299	Undergraduate students	NA	CTS	AAQ	Insecurity (*B* = 0.09)
Rapoza and Baker, 2008	USA	Cross-sectional	Female	171	Couples	19.77 (3.06)	CTS2	Questionnaire created for the study	NSO
			Male	171					
Weston, 2008	USA	Longitudinal	Female	574	Low income community	33.97 (7.73)	SVAWS	RQ modified version	Avoidance (*r* = 0.25)
Wigman et al., 2008	UK	Cross-sectional	Female	127	Undergraduate students	21 (6.41)	CTS	RQ	Security (*r* = −0.16)
			Male	50		25 (8.84)			
Scott and Babcock, 2009	USA	Cross-sectional	Female	138	In violent relationship	29.74 (9.34)	CTS2	AAS	Anxiety (*F* = 19.85; *p* < 0.001)
			Female	37	In non-violent relationship	31.51 (10.17)			
Kuijpers et al., 2012	The Netherlands	Longitudinal	Female	74	Help-seeking victims of IPV	39.28 (10.04)	CTS2	ECR-S	Avoidance (*r* = 0.43)
Gay et al., 2013	USA	Cross-sectional	Female	396	College students	19.14 (1.4)	CTS2	RSQ	Anxiety (*r* = 0.14) Avoidance (*r* = 0.10)
Karakurt et al., 2013	USA	Cross-sectional	Female	87	Couples	22.3 (4.80)	CTS	ECR	Insecurity (*r* = 0.39 for M)
			Male	87					
Owens et al., 2014	USA	Cross-sectional	Male	133	Veterans with PTSD	51.28 (12.05)	CTS	ECR-S	Anxiety (*r* = 0.19)
Craparo et al., 2014	Italy	Cross-sectional	Female	80	Victims of IPV	31.62 (9.81)	None	ASQ	Confidence (*F* = 11.82; *p* < 0.05) Discomfort (*F* = 20.16 *p* < 0.05) Need for Approval (*F* = 4.97; *p* < 0.05) Preoccupation (*F* = 10.57; *p* < 0.05)
			Female	80	Not victims of IPV	25.05 (3.67)			
Oka et al., 2014	USA	Cross-sectional	Female	644	Couples	30.25 (9.79)	3 items of the CTS2	BARE	Insecure (*b* = 0.16)
			Male	644		32.44 (10.5)			
Hellemans et al., 2015	Belgium	Cross-sectional	Female	392	Turkish minority in Belgium	34.32 (10.74)	7 items adopted from the WHO'study	ECR-S	Avoidance (*r* = 0.25)
Bélanger et al., 2015	Canada	Cross-sectional	Male	23	Help-seeking abusive men	34.3 (NA)	CTS2	ECR-S	Avoidant (*r* = 0.53)
Karakoç et al., 2015	Turkey	Cross-sectional	Female	36	Depressive patients	39.10 (10.2)	Questionnaire created for the study	ASQ	Anxiety (*t* = 3.9; *p* < 0.05) Avoidance (*t* = 2.8; *p* < 0.05)
				64	Depressive patients with history of IPV				
Seiffge-Krenke and Burk, 2015	Germany	Cross-sectional	Female	194	Couples	16.99 (1.26)	CADRQ	ECR	Anxiety (*r* = 0.21 for M; *r* = 0.27 for F)
			Male	194		18.41 (2.02)			
Smith and Stover, 2016	USA	Longitudinal	Female	93	With history of IPV	30 (NA)	CTS2	ECR-R	Anxiety (*r* = 0.32)
González et al., 2016	Chile	Cross-sectional	Female	407	University students	21.36 (4.48)	CTS2	ECR	Anxiety (statistical index NA)
			Male	334		21.38 (2.18)			
Sandberg et al., 2016	USA	Longitudinal	Female	133	College students	22.10 (6.38)	CTS2	ECR	Anxiety (*r* = 0.23)
Bonache et al., 2017	Spain	Cross-sectional	Female	638	Students	15.41 (1.11)	CTS	ECR-R	Avoidance (*r* = 0.19)
			Male	660					
Smagur et al., 2018	USA	Cross-sectional	Female	206	Pregnant women with an history of IPV	25.38 (5.00)	SVAWS	ASQ	Anxiety (*r* = 0.21) Avoidance (*r* = 0.37)
Sommer et al., 2017	USA	Cross-sectional	Female	163	Couples	30.29 (9.61)	CTS2	AAS	Anxiety (*r* = 0.17 for F; *r* = 0.23 for M) Avoidance (*r* = 0.23 for F; *r* = 0.31 for M)
			Male	163		31.90 (9.51)			

*IPV, Intimate Partner Violence; NA, Not Available; CTS2, Conflict Tactics Scale Revised; RQ, Relationship Questionnaire; NSO, No Significant Outcome; MSI-R, Marital Satisfaction Inventory revised; F, Female; M, Male; PAPS, Physical Abuse of Partner Scale; HAI, History of Attachment Interview; ECR, The Experiences of Close Relationship Questionnaire; ECR-R, Experiences of Close Relationship Questionnaire Revised; HAQ, History of Abuse Questionnaire; AAQ, Adult Attachment Questionnaire; RSQ, Relationship Style Questionnaire; ABI, Abusive Behavior Inventory; ASQ, Attachment Style Questionnaire; SVAWS, Severity of Violence Against Women Scale; TLEQ, Traumatic Life Events Questionnaire; ECR-S, Experiences in Close Relationships Questionnaire Short Form; AAS, Adult Attachment Scale; BARE, Brief Accessibility, Responsiveness and Engagement Scale; CADRQ, Conflict in Adolescent Dating Relationships Questionnaire*.

Eight studies were conducted on women with a previous reported history of IPV whereas more than a third of the studies used students as participants. Seven studies were conducted on couples whereas 15 groups of researchers focused exclusively on female population. Interestingly, two studies examined the topic among clinical population suffering from PTSD and depression.

The Conflict Tactics Scale (CTS, Straus, 1979) and its revised version (CTS2, Straus et al., 1996) were the most used instrument for the assessment of physical IPV (65.52% of the studies). Instruments measuring attachment were more heterogeneous with the ECR being the most used (44.83% of the studies).

Noteworthy, all studies merging the avoidance and anxiety dimensions into a unique insecure one, evidenced a positive and significant association with physical victimization (Toews et al., 2005; Higginbotham et al., 2007; Karakurt et al., 2013). Interestingly, whereas these first evidences were brought by two studies focusing exclusively on females, Karakurt et al. (2013) successively found that such association was significant only among male participants.

Considering the specific dimensions of attachment, only four studies failed to find some kind of relationship with physical IPV victimization (Bookwala, 2002; Orcutt et al., 2005; Shurman and Rodriguez, 2006; Rapoza and Baker, 2008). In relation to the anxious facet of attachment, results are highly contrasting with 14 studies finding a role played by this dimension in physical IPV victimization and other 10 failing to replicate such result. Correlational studies providing support to the hypothesis of a relationship between physical IPV victimization and attachment reported coefficients ranging from 0.14 to 0.42. Also, studies conducted on clinical participants suffering from PTSD or depression are in line with these results (Owens et al., 2014; Karakoç et al., 2015).

Regarding the avoidant dimension, again, studies are split in two balanced categories of results with 14 failing to find any association with physical IPV victimization and 11 pointing out a significant relationship between the two variables. However, it has to be noted that from the last category, three studies suffer from methodological concerns related to the lack of a validated measure of IPV (Craparo et al., 2014; Hellemans et al., 2015; Karakoç et al., 2015). Anyway, the intensity of the significant associations reported vary from 0.10 to 0.53 with one study not reporting any statistical index (Rogers et al., 2005). Interestingly, the strongest correlation was obtained among a sample of men being in treatment for IPV perpetration (Bélanger et al., 2015).

Some studies did not limit the investigation to the relationship between attachment and IPV victimization but add other insightful considerations. For example, several studies tested this relationship considering the role of early trauma. First, results brought by Sandberg et al. (2016) underlined that anxious attachment significantly predicted physical IPV victimization also after controlling the role of trauma. In contrast, Karakoç et al. (2015) showed that, when controlling for the effect of trauma, insecure attachment no longer predicted IPV victimization among patients suffering from depression. Also, Smith and Stover (2016) found that childhood maltreatment predicted IPV victimization only when participants scored high on the anxious dimension of attachment. However, Gay et al. (2013) showed that insecure attachment did not mediate the relationship between childhood maltreatment and IPV victimization. No other studies tested such mediation model. Then, insecure attachment has been showed to moderate the link between IPV victimization and PTSD symptoms (Scott and Babcock, 2009) and to mediate the relationship between physical victimization and depressive symptomatology (Smagur et al., 2018). Furthermore, others variables seem to play a role in the relationship between attachment and IPV victimization as conflict resolution strategies (Bonache et al., 2017), anger (Kuijpers et al., 2012) and religiosity (Higginbotham et al., 2007). Finally, gender differences emerged in the study of Hellemans et al. (2015) suggested that physical IPV victimization was related with attachment avoidance among women and with attachment anxiety among men.

#### Psychological IPV victimization and attachment

Since 1997, 23 studies investigating attachment among victims of psychological IPV have been published. Characteristics of these studies are illustrated in Table 3.

**Table 3:** Studies investigating the relationship between psychological IPV and attachment among victims.

**References**	**Country**	**Design**	**Sample characteristics**				**Instrument used to evaluate IPV**	**Instrument used to evaluate attachment**	**Main results**
			**Gender composition**	**Size**	**Type**	**Age**			
Henderson et al., 1997	Canada	Longitudinal	Female	63	With history of IPV	31.4 (NA)	CTS	Interview coded with the Bartholomew's model	NSO
O'Hearn and Davis, 1997	USA	Cross-sectional	Female	282	Undergraduate students	NA	PMWI Interview	RQ Interview	For self-report: NSO For Interview: Security (*r* = −0.35) Preoccupied (*r* = 0.33)
Henderson et al., 2005	Canada	Cross-sectional	Male and Female	128	Community participants	37.4 (12.6)	CTS2	HAI	Preoccupied (*r* = 0.38)
Toews et al., 2005	USA	Cross-sectional	Female	147	Divorced mothers	34 (NA)	CTS2	RSQ	Insecurity (*r* = 0.31)
Shurman and Rodriguez, 2006	USA	Cross-sectional	Female	85	Help-seeking victims of IPV	33.89 (9.6)	ABI	ASQ	NSO
Weston, 2008	USA	Longitudinal	Female	574	Low income community	33.97 (7.73)	SOPAS	RQ modified version	Avoidant (*r* = 0.37 for insecure F; *r* = 0.35 for secure F)
Wigman et al., 2008	UK	Cross-sectional	Female	127	Undergraduate students	21 (6.41)	CTS	RQ	NSO
			Male	50		25 (8.84)			
Riggs and Kaminski, 2010	USA	Cross-sectional	Female	213	College students	21.9 (3.70)	CTS2	ECR	Anxiety (*r* = 0.15)
			Male	64		21.4 (4.25)			
Péloquin et al., 2011	Canada	Cross-sectional	Female	193	Couples	31 (NA)	CTS2	ECR	Anxiety (*r* = 0.19 for F; *r* = 0.32 for M) Avoidance (*r* = 0.29 for F)
			Male	193					
Kuijpers et al., 2012	The Netherlands	Longitudinal	Female	74	Help-seeking victims of IPV	39.28 (10.04)	CTS2	ECR-S	Avoidance (*r* = 0.32)
Karakurt et al., 2013	USA	Cross-sectional	Female	87	Couples	22.3 (4.80)	EAQ	ECR	Insecurity (*r* = 0.43 for M)
			Male	87					
Owens et al., 2014	USA	Cross-sectional	Male	133	Veterans with PTSD	51.28 (12.05)	CTS	ECR-S	Anxiety (*r* = 0.22) Avoidance (*r* = 0.26)
Oka et al., 2014	USA	Cross-sectional	Female	644	Couples	30.25 (9.79)	3 items of the CTS2	BARE	NSO
			Male	644		32.44 (10.5)			
Hellemans et al., 2015	Belgium	Cross-sectional	Female	392	Turkish minority in Belgium	34.32 (10.74)	7 items adopted from the WHO'study	ECR-S	Anxiety (*r* = 0.22) Avoidance (*r* = 0.37)
Bélanger et al., 2015	Canada	Cross-sectional	Male	23	Help-seeking abusive men	34.3 (NA)	CTS2	ECR-S	NSO
Seiffge-Krenke and Burk, 2015	Germany	Cross-sectional	Female	194	Couples	16.99 (1.26)	CADRQ	ECR	Anxiety (*r* = 0.24 for M; *r* = 0.32 for F) Avoidance (*r* = 0.20 for M; *r* = 0.22 for F)
			Male	194		18.41 (2.02)			
Tougas et al., 2016	Canada	Cross-sectional	Female	210	Couples	41 (NA)	CTS2	ECR	NSO
			Male	210		43 (NA)			
Bonache et al., 2016	Spain	Cross-sectional	Female	165	Undergraduate students	21.40 (3.63)	CIRS	ECR-R	Anxiety (*r* = 0.58) Avoidance (*r* = 0.50)
			Male	51					
Goncy and van Dulmen, 2016	USA	Cross-sectional	Female	113	Dating non married couples	19.13 (0.80)	CADRI	ECR-R	Anxiety (*r* = 0.37 for F; *r* = 0.38 for M) Avoidance (*r* = 0.20 for F; *r* = 0.22 for M)
			Male	113		20.25 (1.80)			
Oka et al., 2016	USA	Cross-sectional	Female	457	Couples	43.8 (NA)	CRAVIS	ECR	Insecurity (*r* = 0.53 for F, *r* = 0.48 for M)
			Male	457		45.6 (NA)			
Bonache et al., 2017	Spain	Cross-sectional	Female	638	Students	15.41 (1.11)	SDPAV	ECR-R	Anxiety (*r* = 0.27) Avoidance (*r* = 0.23)
			Male	660					
Sommer et al., 2017	USA	Cross-sectional	Female	163	Couples	30.29 (9.61)	CTS2	AAS	Anxiety (*r* = 0.17) Avoidance (*r* = 0.23 for F; *r* = 0.31 for M)
			Male	163		31.90 (9.51)			
Smagur et al., 2018	USA	Longitudinal	Female	206	Pregnant and with history of IPV	25.38 (5.00)	SVAWS	ASQ	Anxiety (*r* = 0.21) Avoidance (*r* = 0.37)

*IPV, Intimate partner Violence; NA, Not available; CTS, Conflict Tatics Scale; NSO, No Significant Outcome; PMWI, Psychological Maltreatment of Women Inventory; RQ, Relationship Questionnaire; HAI, History of Attachment Interview; ABI, Abusive Behavior Inventory; ASQ, Attachment Style Questionnaire; SOPAS, Subtle and Overt Psychological Abuse Scale; F, Female; M, Male; CTS2, Conflict Tatics Scale revised; ECR, Experiences in Close Relationship; ECR-S, Experiences in Close Relationship Short Form; EAQ, Emotional Abuse Questionnaire; CRAVIS, Couples Relational Aggression and Victimization Scale; BARE, Brief Accessibility Responsiveness and Engagement scale; CADRQ, Conflict in Adolescent Dating Relationships Questionnaire; RSQ, Relationships Style Questionnaire; CIRS, Conflict Resolution Styles Inventory; ECR-R, Experiences in Close Relationships Revised; CADRI, Conflict in Adolescent Dating Relationships Inventory; SDPAV, Safe Dates-Psychological Abuse Victimization; AAS, Adult Attachment Scale; SVAWS, Severity of Violence Against Women Scale*.

Among them, 54.54% were conducted in USA, 22.72% in Europe and 18.18% in Canada. Despite the fact that the very two first studies were published in 1997, 81.81% of them have been published in the last 10 years. Only 18.18% of the studies were longitudinal in their design with the others being cross-sectional. Regarding sample types, most of the researches were conducted on couples (Péloquin et al., 2011; Karakurt et al., 2013; Oka et al., 2014, 2016; Seiffge-Krenke and Burk, 2015; Goncy and van Dulmen, 2016; Tougas et al., 2016; Sommer et al., 2017). Fortunately, only five groups of researchers used student samples (O'Hearn and Davis, 1997; Wigman et al., 2008; Riggs and Kaminski, 2010; Bonache et al., 2016, 2017). Two additional studies were conducted among male-only samples being veterans suffering from PTSD (Owens et al., 2014) or batterers (Bélanger et al., 2015). Unfortunately, only a small proportion of studies recruited women reporting experiences of psychological IPV (Henderson et al., 1997; Shurman and Rodriguez, 2006; Kuijpers et al., 2012; Smagur et al., 2018). Finally, two studies examined the topic among minority populations of women (Weston, 2008; Hellemans et al., 2015). Instruments used to evaluate both IPV and attachment were homogenous with ECR being mostly used to evaluate attachment styles and the subscale of CTS used to measure the intensity of psychological IPV.

Three studies, merging the anxiety and avoidance dimensions in a unique index of insecure attachment, found that psychological IPV victimization was positively correlated with insecure attachment (Toews et al., 2005; Karakurt et al., 2013; Oka et al., 2016) with coefficient ranging from 0.31 to 0.53. Noteworthy, almost half of the studies found that psychological IPV victimization was not associated with anxious or avoidant dimensions of attachment (Henderson et al., 1997; Shurman and Rodriguez, 2006; Wigman et al., 2008; Oka et al., 2014; Bélanger et al., 2015; Tougas et al., 2016).

Regarding the anxious dimension, studies conducted on women with reported history of IPV mainly failed to find an association between anxious attachment and psychological victimization (Henderson et al., 1997; Shurman and Rodriguez, 2006; Kuijpers et al., 2012). In contrast, studies recruiting students or community participants mostly indicated a relationship between anxious attachment and IPV among victims, suggesting a potential role played by sample type (O'Hearn and Davis, 1997; Henderson et al., 2005; Riggs and Kaminski, 2010; Bonache et al., 2016, 2017). Noteworthy, such studies greatly vary in the intensity of reported association with correlational coefficients ranging from 0.15 to 0.58. Finally, whereas some studies conducted on couples reported association between psychological IPV and anxious attachment among victims (Péloquin et al., 2011; Seiffge-Krenke and Burk, 2015; Goncy and van Dulmen, 2016; Sommer et al., 2017), two others studies failed to replicate such results (Oka et al., 2014; Tougas et al., 2016). However, the study of Oka et al. (2014) may be biased by methodological issues as IPV was measured throughout only three items extracted from the Conflict Tactics Scale-Revised.

Then, from 20 studies examining the relationship between avoidant attachment and psychological IPV, only 11 found a significant association between the constructs with correlational coefficients ranging from 0.20 to 0.50. In relation to the role played by gender in such relationship, Péloquin et al. (2011) found that this association was significant only among females whereas two other studies indicated significant correlations in both gender (Seiffge-Krenke and Burk, 2015; Goncy and van Dulmen, 2016; Sommer et al., 2017).

Some studies shed light on additional interesting aspects related to the link between attachment and psychological IPV victimization. First, gender differences emerged in some studies (Péloquin et al., 2011; Hellemans et al., 2015). Also, studies showed that insecure attachment not only predicted psychological IPV victimization beyond the role of depression (Riggs and Kaminski, 2010) but also mediated such relationship (Smagur et al., 2018). Finally, the use of destructive conflict strategies has been showed to explain the pathway by which insecure attachment leads to psychological IPV victimization (Bonache et al., 2016, 2017).

#### Sexual IPV victimization and attachment

Only six studies investigated the relationship between sexual IPV and attachment among victims (see Table 4). Interestingly, these studies are relatively recent with the majority having been published in the last 3 years. All of them, except one (Bonache et al., 2016), were conducted in USA (Weston, 2008; Karakurt et al., 2013; Ross et al., 2016; Sommer et al., 2017; Smagur et al., 2018). Half of the studies were cross-sectional in their design with the remainders being longitudinal. Studies recruited large samples ranging from 51 to 574 participants by group (Weston, 2008; Bonache et al., 2016). Noteworthy, only one research was conducted on participants with a reported history of IPV (Smagur et al., 2018) with most of others recruiting couples extracted from general population (Karakurt et al., 2013; Sommer et al., 2017) or undergraduate students (Bonache et al., 2016; Ross et al., 2016).

**Table 4:** Studies investigating the relationship between sexual IPV and attachment among victims.

**References**	**Country**	**Design**	**Sample Characteristics**				**Instrument used to evaluate IPV**	**Instrument used to evaluate attachment**	**Main results**
			**Gender composition**	**Size**	**Type**	**Age**			
Weston, 2008	USA	Longitudinal	Female	574	Low income community	33.97 (7.73)	SVAWS	RQ	Avoidance (*r* = 0.22 for secure F; *r* = 0.14 for insecure F)
Karakurt et al., 2013	USA	Cross-sectional	Female	87	Couples	22.3 (4.80)	CTS	ECR	NSO
			Male	87					
Bonache et al., 2016	Spain	Cross-sectional	Female	165	Undergraduate students	21.40 (3.63)	SCIRS	ECR-R	Anxiety (*r* = 0.53) Avoidance (*r* = 0.34)
			Male	51					
Ross et al., 2016	USA	Longitudinal	Female	584	Undergraduate Students	20.43 (4.64)	SCIRS	ECR-S	Anxiety (*F* = 3.11, *p* < 0.05)
			Male	301					
Sommer et al., 2017	USA	Cross-sectional	Female	163	Couples	30.29 (9.61)	CTS2	AAS	Anxiety (*r* = 0.17 for F; *r* = 0.24 for M) Avoidance (*r* = 0.23 for M)
			Male	163		31.90 (9.51)			
Smagur et al., 2018	USA	Longitudinal	Female	206	Pregnant and with history of IPV	25.38 (5.00)	SVAWS	ASQ	Anxiety (*r* = 0.26) Avoidance (*r* = 0.39)

*IPV, Intimate Partner Violence; SVAWS, Severity of Violence Against Women Scale; RQ, Relationship Questionnaire; F, Female; CTS, Conflict Tactics Scale; ECR, Experience in Close Relationship Questionnaire; NSO, No Significant Outcome; SCIRS, Sexual Coercion Victimization Scale; ECR-R, Experience in Close Relationship Questionnaire Revised; ECR-S, Experiences in Close Relationship Questionnaire Short Form; AAS, Adult Attachment Scale; M, Male; ASQ, Attachment Style Questionnaire*.

Results regarding the relationship between the anxious dimension of attachment among victims of sexual IPV are contrasting. Some found that sexual IPV victimization correlated positively and significantly with anxiety (Sommer et al., 2017; Smagur et al., 2018) with coefficient reaching 0.53. Also, results of Ross et al. (2016) indicated that individuals with history of sexual IPV scored higher on the anxious dimension of the Experiences in Close Relationships-Short form (ECR-S, Wei et al., 2007) compared to participants without experiences of sexual IPV. In contrast, two studies failed to find significant association between sexual IPV victimization and anxious attachment (Weston, 2008; Karakurt et al., 2013).

In relation the attachment dimension of avoidance, data brought by correlational studies mostly indicated a positive association between anxious attachment and sexual IPV with coefficients ranging from 0.22 to 0.39. Noteworthy, one of them found that the association was significant only among men. However, two studies did not go in the same direction, finding no association between avoidance and sexual IPV victimization (Karakurt et al., 2013) or no differences on avoidance scores between individuals with and without sexual IPV victimization (Ross et al., 2016).

Finally, two studies examined other variables accounting for the relationship between attachment and sexual IPV victimization showing that such link was mediated by the use of destructive conflict resolution strategies (Bonache et al., 2016) and that insecure attachment fully mediated the pathway by which childhood maltreatment leads to sexual IPV victimization (Smagur et al., 2018).

As a whole, research examining the role of attachment in IPV victimization appears widely unbalanced in relation to the type of violence investigated, with most studies measuring physical manifestation and only few including a separate measurement of sexual victimization. Despite the fact that the majority of studies found some kind of association between insecure attachment and IPV victimization, results are highly contrasting regarding the specific dimensions of attachment.

### IPV perpetration and attachment

In the present review, we found 72 studies that explored the attachment dimensions among IPV perpetrators. Contrary to the studies on IPV victimization, most of the studies focused on psychological IPV (40.74%), whereas 15.52% of the studies did not make differences between the different forms of IPV, 31.04% of the studies investigated physical IPV and on 6.79% of the studies were focused on sexual IPV. The studies we examined cover a wide range of years, comprised between 1994 and 2017, even though the majority has been published in the last 10 years.

#### Generic IPV perpetration and attachment

Over the years, 15 studies decided to investigate the relationship between the perpetration of violence in general, not discriminating between different forms of expression, and attachment. These studies are displayed in Table 5.

**Table 5:** Studies investigating the relationship between generic IPV and attachment among perpetrators.

**References**	**Country**	**Design**	**Sample Characteristics**				**Instrument used to evaluate IPV**	**Instrument used to evaluate attachment**	**Main results**
			**Gender composition**	**Size**	**Type**	**Age**			
Babcock et al., 2000	USA	Cross-sectional	Male	23	Violent males	NA	CTS	AAI	Insecure attachment (khi-2; *p* < 0.05)
				13	Non-violent males				
Wigman et al., 2008	UK	Cross-sectional	Male	50	College students	22 (7.39)	UPBI	RQ	Preoccupied (*r* = 0.16) Fearful (*r* = 0.18)
			Female	127					
Carraud et al., 2008	France	Cross-sectional	Male	50	Convicted for IPV	18 (NA)	CTS2	ECR	Preoccupied, dismissing (khi-2 *p* < 0.05)
					Convicted for other crimes				
Grych and Kinsfogel, 2010	USA	Cross-sectional	Male	188	High School students	15.6 (1.1)	CIR	ECR	Anxiety (*r* = 0.19 for M; *r* = 0.17 for F)
			Female	203					
Weiss et al., 2011	USA	Cross-sectional	Male	66	High school students with ID	15.58 (0.98)	CADRI	Attachment Security Ratings	Avoidance (*r* = 0.30)
			Female	90					
De Smet et al., 2012	Belgium	Cross-sectional	Male	160	Divorced	43.1 (9.42)	RP-PSF	ECR-S	Anxiety (khi-2 = 9.58, *p* < 0.01)
			Female	236					
Gay et al., 2013	USA	Cross-sectional	Female	409	College students	19.14 (1.4)	CTS2	RSQ	Anxiety (*r* = 0.11)
De Smet et al., 2013	Belgium	Cross-sectional	Male	46	Former couples	47.07 (8.3)	RP-PSF	ECR-S	Anxiety (*r* = 0.37 for M; *r* = 0.41 for F)
			Female	46		44.8 (7.88)			
Genest and Mathieu, 2014	Canada	Cross-sectional	Male	80	Males in treatment for IPV	34.3 (NA)	CTS2	ECR	NSO
Tassy and Winstead, 2014	USA	Cross-sectional	Male	62	College students	19.5 (NA)	RP-PSF	ECR-S	Anxiety (*r* = 0.19)
			Female	180					
Ulloa et al., 2014	USA	Longitudinal	Male	62	High school students	15.87 (1.52)	CADRI	AAS	Anxiety (*r* = 0.06)
			Female	78					
Muñoz, 2015	Chile	Cross-sectional	Male	732	Males in treatment for IPV	NA	CTS2	ECR-R	Anxiety (*F* = 8.1, *p* = 0.000)
				100	Non-violent males				
Pimentel and Santelices, 2017	Chile	Cross-sectional	Male	20	Violent males	39 (7.7)	CTS2	ECR	Anxiety (*U* = 93.5; *p* < 0.004)
				20	Non-violent males				
Gonzalez-Mendez et al., 2017	Spain	Cross-sectional	Male	166	High school students	15.66 (1.23)	SD CTS-S	ECR-R	Anxiety (*r* = 0.31)
			Female	190					
McClure and Parmenter, 2017	USA	Cross-sectional	Male	93	College students	18.83 (1.03)	CADRI	AAS	NSO
			Female	161					
Aizpitarte et al., 2017	Spain	Cross-sectional	Male	197	High school students	18.02 (1.36)	CADRI	ECR	Anxiety (*r* = 0.33) Avoidance (*r* = 0.13)
			Female	280					

*NA, Not available; CTS, Conflict Tactics Scale; AAI, Adult Attachment Interview; UPBI, Unwanted Pursuit Behavior Inventory; RQ, Relationship Questionnaire; IPV, Intimate Partner Violence; CTS2, Conflict Tactics Scale-Revised; ECR, Experiences in Close Relationships Scale; CIR, Conflict in Relationships scale; CADRI, Conflict in Adolescent Dating Relationships Inventory; F, Female; M, Male; RP-PSF, Relational Pursuit-Pursuer Short Form; ECR-S, Experiences in Close Relationships Scale-Short form; AAS, Adult Attachment Scale; RSQ, Relationship Style Questionnaire; NSO, Non-significant outcome; ECR-R, Experiences in Close Relationships Scale-Revised; SD, Safe Dates; CTS-S, Conflict Tactics Scale-Short form*.

America and Europe have been the continents in which studies were mainly conducted: most of the studies were run in USA (33.3%), followed by Spain (13.3%), Chile (13.3%), and Belgium (13.3%). There was only one studied conducted in France, one in Canada and one in UK.

Research are mainly cross-sectional in their design with only one study adopting a longitudinal design of research (Ulloa et al., 2014).

Only six studies (Babcock et al., 2000; Carraud et al., 2008; Gay et al., 2013; Genest and Mathieu, 2014; Muñoz, 2015; Pimentel and Santelices, 2017) did not investigate the topic among samples balanced for gender.

Regarding sample types, researchers often used participants with no previous report of IPV such as students (Wigman et al., 2008; Grych and Kinsfogel, 2010; Gay et al., 2013; Tassy and Winstead, 2014; Ulloa et al., 2014; Aizpitarte et al., 2017; Gonzalez-Mendez et al., 2017; McClure and Parmenter, 2017) or minority population such as divorced couples (De Smet et al., 2012, 2013) and jail population (Carraud et al., 2008). In this case, only four studies recruited participants with a previous reported history of IPV (Babcock et al., 2000; Genest and Mathieu, 2014; Muñoz, 2015; Pimentel and Santelices, 2017).

Surprisingly, most studies investigated the topic among young adult population and adolescents and only six studies recruited adult population (Babcock et al., 2000; De Smet et al., 2012, 2013; Genest and Mathieu, 2014; Muñoz, 2015; Pimentel and Santelices, 2017).

Due to the age variability of the samples, there was a relevant heterogeneity concerning the instruments used to measure IPV: CTS, both in its revised and its short version (Control Tactics Scale-Short form; CTS-S), has been the most used in adult samples, whereas CADRI was the most used with adolescents. As for attachment measures, ECR, both in its revised (Experiences in Close Relationships-Revised; ECR-R, Fraley et al., 2000) and its short version, turns out to be the most used tool (62.5%) both for adult and for adolescent population. Also, an American study conducted in 2000 used Adult Attachment Interview (AAI; George et al., 1985) on batterers.

Results of the studies supported the hypothesis of a relationship between attachment dimensions and being a perpetrator of IPV. All the studies that confronted a clinical group of violent men with a control group of non-violent men (Babcock et al., 2000; Carraud et al., 2008; Muñoz, 2015; Pimentel and Santelices, 2017) proved that violent men tend to have insecure attachment (Babcock et al., 2000), showing a higher level of anxiety in close relationships (Muñoz, 2015; Pimentel and Santelices, 2017) compared to non-violent men, even though results do not agree with each other about the prevailing attachment style of clinical groups. There has been found a prevalence of preoccupied and dismissing attachment (Carraud et al., 2008) over other attachment styles.

Most of the studies support the existence of a positive correlation between the attachment dimension of anxiety and IPV perpetration, even though coefficient did not indicate any strong association. They ranged from 0.06 to 0.33.

Both the weak correlation of 0.06 and a study that did not obtain any significant outcome (McClure and Parmenter, 2017) may be explained by the use of the Adult Attachment Scale (AAS, Hazan and Shaver, 1987), which might be not enough sensitive as a tool for this specific target group, as claimed by Tasso et al. (2012).

Concerning the attachment dimension of avoidance, only two studies found a significant correlation between IPV perpetration and avoidance (Weiss et al., 2011; Aizpitarte et al., 2017), where other studies failed to replicate the same result. Correlational coefficients range from 0.30 to 0.13: as for anxiety dimension scores indicate a weak association between the two constructs.

#### Physical IPV perpetration and attachment

Since 1998, the relationship between attachment and physical IPV as perpetrators has been investigated in 32 studies, illustrated in Table 6.

**Table 6:** Studies investigating the relationship between physical IPV and attachment among perpetrators.

**References**	**Country**	**Design**	**Sample Characteristics**				**Instrument used to evaluate IPV**	**Instrument used to evaluate attachment**	**Main results**
			**Gender composition**	**Size**	**Type**	**Age**			
Wekerle and Wolfe, 1998	Canada	Cross-sectional	Male	128	High school students	15.34 (1.75)	CIR	Attachment Security Ratings	Anxiety (*r* = 0.15) Avoidance (*r* = 0.21)
			Female	193		15.13 (0.94)			
Rankin et al., 2000	USA	Cross-sectional	Male	69	Convicted for IPV	31 (11)	MWA	ASQ	Avoidance (*r* = 0.27)
Follingstad et al., 2002	USA	Cross-sectional	Male	223	College students	NA	CTS	RSQ	NSO
			Female	199					
Kim and Zane, 2004	USA	Cross-sectional	Male	52	Korean Americans in treatment for IPV	40.7 (9.8)	CTS	RQ	Anxiety (*r* = 0.28)
				50	European Americans in treatment for IPV				
Henderson et al., 2005	Canada	Cross-sectional	Male	60	Community	37.4 (12.6)	CTS2	HAI	Preoccupied (*r* = 0.23)
			Female	68					
Orcutt et al., 2005	USA	Cross-sectional	Female	457	College students	NA	CTS2	ECR-R	Anxiety (*b* = 0.32) Avoidance (*b* = −0.11)
Lafontaine and Lussier, 2005	Canada	Cross-sectional	Couples	316	Community	39 (NA)	CTS2	ECR	Anxiety (β = .15 for F)
Toews et al., 2005	USA	Cross-sectional	Female	147	Divorced mothers	34 (NA)	CTS2	RSQ	Insecure attachment (*r* = 0.25)
Lawson et al., 2006	USA	Cross-sectional	Male	33	In treatment for IPV	32.8 (8.7)	CTS	AAS	NSO
Mauricio et al., 2007	USA	Cross-sectional	Male	192	Convicted for IPV	33 (8.83)	CTS	ECR	Anxiety (*r* = 0.24)
Goldenson et al., 2007	USA	Cross-sectional	Female	33	Violent	30.9 (7.8)	Physical violence interview	ECR-R	Anxiety (*F* = 8.48, *p* = 0.005) Avoidance (F = 10.96, *p* = 0.002)
				32	Non-violent	32 (9.1)			
Lawson, 2008	USA	Cross-sectional	Male	100	Violent	32.2 (10.3)	CTS	AAS	Closeness (*r* = −0.32)
				35	Non-violent	27.1 (10)			
Wigman et al., 2008	UK	Cross-sectional	Male	50	College students	22 (7.39)	CTS	RQ	NSO
			Female	127					
Doumas et al., 2008	USA	Cross-sectional	Male	70	Community couples	28.46 (10.36)	CTS	RQ	NSO
			Female	70		27.03 (10.52)			
Godbout et al., 2009	Canada	Cross-sectional	Male	315	Community	29.5 (5.5)	CTS2	ECR	Anxiety (*r* = 0.21) Avoidance (*r* = 0.15)
			Female	329		27.6 (4.3)			
Lawson and Brossart, 2009	USA	Longitudinal	Male	49	In IPV treatment	31.73 (8.83)	CTS2	AAS	NSO
Brown et al., 2010	Australia	Cross-sectional	Male	66	In IPV treatment	39.9 (NA)	ABI	SSDS	Anxiety (*r* = 0.27)
Miga et al., 2010	USA	Longitudinal	Male	39	Couples	14.28 (0.78)	CIR	ECR	NSO
			Female	54					
Lawson and Malnar, 2011	USA	Cross-sectional	Male	100	On probation for IPV	32.2 (10.3)	MCTS	AAS	Avoidant (*r* = 0.24)
Fournier et al., 2011	Canada	Cross-sectional	Male	55	In treatment for relationship difficulties	37 (12.5)	CTS2	ECR	Anxiety (*r* = 0.32)
Karakurt et al., 2013	USA	Cross-sectional	Male	87	Couples of college students	22.3 (4.8)	CTS2	ECR; RQ	NSO
			Female	87					
Owens et al., 2014	USA	Cross-sectional	Male	133	Veterans in treatment for PTSD	51.28 (12.05)	CTS	ECR-S	Anxiety (*r* = 0.19)
McKeown, 2014	UK	Cross-sectional	Female	92	Convicted	NA	CTS2	ECR-R	NSO
Brassard et al., 2014	Canada	Cross-sectional	Male	302	In treatment for relationship difficulties	35 (10.9)	CTS2	ECR	Anxiety (*r* = 0.19)
Lee et al., 2014	USA	Cross-sectional	Male	89	College students	20.81 (1.81)	CTS2	ECR-R	Anxiety (*r* = 0.34)
			Female	392					
Belus et al., 2014	USA	Cross-sectional	Male	125	College students	21.25 (2.21)	CTS2	RSQ	NSO
			Female	306		21.39 (3.6)			Fearful (*r* = 0.13) Secure (*r* = −0.12) Preoccupied (*r* = 0.13)
Burk and Seiffge-Krenke, 2015	Germany	Cross-sectional	Male	194	Couples of high school students	16.99 (1.26)	CADRI	ECR	Anxiety (*r* = 0.37 for M; *r* = 0.19 for F) Avoidance (*r* = 0.30 for M)
			Female	194		18.41 (2.02)			
Rodriguez et al., 2015	USA	Cross-sectional	Male	39	College students	22.51 (4.79)	CTS2	ECR	Anxiety (*r* = 0.27)
			Female	222					
Bélanger et al., 2015	Canada	Cross-sectional	Male	20	Couples in treatment for IPV	34.3 (NA)	CTS2	ECR	Anxiety (*r* = 0.565 for F)
			Female	20		32.2 (NA)			
González et al., 2016	Chile	Cross-sectional	Male	239	College students	21.52 (2.15)	CTS2	ECR	Anxiety (*r* = 0.14 for M; *r* = 0.12 for F) Avoidance (*r* = 0.15 for M; *r* = 0.12 for F)
			Female	369		21.41 (2.26)			
Sommer et al., 2017	USA	Cross-sectional	Male	163	Couples	31.9 (9.51)	CTS2	AAS	Anxiety (*r* = 0.1666 for M; *r* = 0.165 for F) Avoidance (*r* = 0.306 for M; *r* = 0.227 for F)
			Female	163		30.29 (9.61)			
Cascardi et al., 2017	USA	Cross-sectional	Male	185	College students	19.5 (NA)	CADRI	RSQ	Anxiety (*r* = 0.16)
			Female	327					

*CIR, Conflict in Relationships scale; IPV, Intimate Partner Violence; MWA, Measurement of Wife Abuse; ASQ, Attachment Style Questionnaire; NA, Not available; CTS, Conflict Tactics Scale; RSQ, Relationship Style Questionnaire; NSO, Non-significant outcome; RQ, Relationship Questionnaire; CTS2, Conflict Tactics Scale-Revised; HAI, History of Attachments Interview; ECR-R, Experiences in Close Relationships Scale-Revised; ECR, Experiences in Close Relationships Scale; F, Female; AAS, Adult Attachment Scale; ABI, Abusive Behavior Inventory; SSDS, Spouse-specific dependency scale; MCTS, Modified Conflict Tactics Scale; ECR-S, Experiences in Close Relationship-Short form; CADRI, Conflict in Adolescent Dating Relationships Inventory; LEQ, Love Experiences Questionnaire; M, Male*.

Most of the studies have been conducted in America: in USA (62.5%) and Canada (21.87%). Only three studies have been conducted in Europe, two in UK and one in Germany. Other two studies have been conducted respectively in Chile and in Australia.

In this group of studies, the most common design is cross-sectional with only one study adopting a longitudinal design, performed in USA (Lawson and Brossart, 2009).

Among all the studies, 23 of them investigated the relationship between the two dimensions among samples balanced for gender. Only four studies had female-only samples and 11 had only male samples.

Contrary to what one might thing, only ten studies (Rankin et al., 2000; Kim and Zane, 2004; Lawson et al., 2006; Goldenson et al., 2007; Mauricio et al., 2007; Lawson, 2008; Lawson and Brossart, 2009; Brown et al., 2010; Lawson and Malnar, 2011; Bélanger et al., 2015) recruited participants that had a previous history of IPV or that are convicted or in therapy because of it. Most of the samples are made up of community population: a relevant number of researches enrolled high school or college students whereas others recruited veterans in treatment for PTSD (Owens et al., 2014), men in treatment for relationship issues (Fournier et al., 2011; Brassard et al., 2014), divorced mothers (Toews et al., 2005), and female prisoners (McKeown, 2014).

All studies, except two (Wekerle and Wolfe, 1998; Burk and Seiffge-Krenke, 2015), enrolled adults or young adults in their samples, so the age of participants is quite homogeneous.

Due to this homogeneity, we can observe quite an accordance in the choice of the physical IPV measure: most of the studies used CTS, both in its revised and its short version, whereas three studies used CADRI to assess adolescents. Concerning attachment assessment, there is much more heterogeneity with most studies making use of ECR, both in its revised and its short version.

The hypothesis of a relationship between physical IPV perpetration and attachment dimensions has been supported by the results of the studies. Compared to non-violent samples, physical IPV perpetrators show higher level of anxiety and avoidance (Goldenson et al., 2007) and a preoccupied attachment style (Henderson et al., 2005). Most of the studies supported a positive correlation between physical IPV perpetration and the attachment dimension of anxiety, even though coefficient ranged from 0.56 to 0.12, so they're not really strong.

Concerning the attachment dimension of avoidance, several studies found a positive correlation with physical IPV perpetration. Correlational coefficients range from 0.30 to 0.12, so, as for attachment anxiety, they indicate a weak correlation between the constructs.

Concerning the dimension of closeness, one study found a negative correlation (*r* = −0.32) with physical IPV (Lawson, 2008).

Regarding gender differences, they are consistent with the trend, showing a prevalence of anxiety and avoidance in both male and female physical IPV perpetrators (González et al., 2016; Sommer et al., 2017).

Noteworthy, eight studies didn't obtain any significant outcome. However, two of them used the AAS as attachment measure, which is claimed to be not much sensitive for such samples by Tasso et al. (2012).

#### Psychological IPV and attachment

There are 42 studies that investigated the relationship between attachment and psychological IPV, focusing on IPV perpetration (see Table 7).

**Table 7:** Studies investigating the relationship between psychological IPV and attachment among perpetrators.

**References**	**Country**	**Design**	**Sample characteristics**				**Instrument used to evaluate IPV**	**Instrument used to evaluate attachment**	**Main results**
			**Gender composition**	**Size**	**Type**	**Age**			
Dutton et al., 1994	Canada	Cross-sectional	Male	120	In treatment for IPV	35 (NA)	PMWI	RSQ; RQ	Anxiety (*r* = 0.26)
				40	Non-violent				
Dutton, 1995	Canada	Cross-sectional	Male	140	In treatment for IPV	35 (NA)	PMWI	RSQ	Fearful (*r* = 0.53)
				44	Non-violent				
Dutton et al., 1996	Canada	Cross-sectional	Male	120	In treatment for IPV	35 (NA)	PMWI	RSQ	Fearful (NA)
				40	Non-violent				
O'Hearn and Davis, 1997	USA	Cross-sectional	Female	282	College students	20 (NA)	Verbal abuse subscale (PMWI)	RQ	Preoccupied (*r* = 0.39) Fearful (*r* = 0.14)
Wekerle and Wolfe, 1998	Canada	Cross-sectional	Male	128	High school students	15.34 (1.75)	CIR	Attachment Security Ratings	Anxiety (*r* = 0.17) Avoidance (*r* = 0.24)
			Female	193		15.13 (0.94)			
Rankin et al., 2000	USA	Cross-sectional	Male	69	Convicted for IPV	31 (11)	MWA	ASQ	Avoidance (*r* = 0.28)
Davis et al., 1998	USA	Cross-sectional	Male	46	College students	19 (NA)	PMP	ECR	Anxiety (*r* = 0.25)
			Female	123					
Davis et al., 2002	USA	Cross-sectional	Male	93	College students	19 (NA)	PMP	ECR	Anxiety (*r* = 0.22)
			Female	110					
Dye and Davis, 2003	USA	Cross-sectional	Male	87	College students	21 (3.31)	PMWI	ECR	Anxiety (*r* = 0.28)
			Female	251					
Henderson et al., 2005	Canada	Cross-sectional	Male	60	Community	37.4 (12.6)	PMWI	HAI	Preoccupied (*r* = 0.38)
			Female	68					
Mahalik et al., 2005	USA	Cross-sectional	Male	143	In treatment for IPV	34.9 (8.99)	CBI	RQ	Fearful (*r* = 0.28)
Lafontaine and Lussier, 2005	Canada	Cross-sectional	Male	316	Couples	39 (NA)	CTS2	ECR	Avoidance (*B* = 0.12 for M) Anxiety (*B* = 0.2 for F)
			Female						
Toews et al., 2005	USA	Cross-sectional	Female	147	Divorced mothers	34 (NA)	CTS2	RSQ	Attachment insecurity (*r* = 0.32)
Mauricio et al., 2007	USA	Cross-sectional	Male	192	In treatment for IPV	33 (8.83)	CTS	ECR	Anxiety (*r* = 0.50) Avoidance (*r* = 0.16)
Lawson, 2008	USA	Cross-sectional	Male	100	Violent	32.2 (10.3)	CTS	AAS	Closeness (*r* = −0.30)
				35	Non-violent	27.1 (10)			
Wigman et al., 2008	UK	Cross-sectional	Male	50	College students	22 (7.39)	CTS	RQ	NSO
			Female	127					
Godbout et al., 2009	Canada	Cross-sectional	Male	315	Community	29.5 (5.5)	CTS2	ECR	Anxiety (*r* = 0.21) Avoidance (*r* = 0.15)
			Female	329		27.6 (4.3)			
Lawson and Brossart, 2009	USA	Longitudinal	Male	49	In treatment for IPV	31.73 (8.83)	CTS2	AAS	NSO
Grych and Kinsfogel, 2010	USA	Cross-sectional	Male	188	High School students	15.6 (1.1)	CIR	ECR	Anxiety (*r* = 0.21 for M; *r* = 0.27 for F)
			Female	203					
Gormley and Lopez, 2010	USA	Cross-sectional	Male	61	College students	20 (2.26)	DS	ECR-R	Anxiety (*r* = 0.30 for F) Avoidance (*r* = 0.48 for M)
			Female	66					
Patton et al., 2010	USA	Cross-sectional	Male	1.169	College students	23 (NA)	NVAWS	ECR	Anxiety (*B* = 0.569)
			Female	1.614					
Brown et al., 2010	Australia	Cross-sectional	Male	66	In treatment for IPV	39.9 (NA)	ABI	SSDS	Anxiety (*r* = 0.39)
Riggs and Kaminski, 2010	USA	Cross-sectional	Male	64	College students	21.4 (4.25)	CTS2	ECR	Anxiety (*r* = 0.160)
			Female	221		21.9 (3.7)			
Péloquin et al., 2011	Canada	Cross-sectional	Male	193	Community couples	31 (NA)	CTS2	ECR	Anxiety (*r* = 0.245 for M; *r* = 0.306 for F) Avoidance (*r* = 0.290 for F)
			Female						
Clift and Dutton, 2011	USA	Cross-sectional	Female	914	College students	20.5 (2.7)	PMI	RSQ	NSO
Fournier et al., 2011	Canada	Cross-sectional	Male	55	In treatment for relationship difficulties	37 (12.5)	CTS2	ECR	Anxiety (*r* = 0.37) Avoidance (*r* = 0.407)
Frey et al., 2011	USA	Cross-sectional	Male	40	Veterans in treatment for PTSD	28.5 (5.11)	IJS	MIMARA	Avoidance (*r* = 0.653 for M)
Lawson and Malnar, 2011	USA	Cross-sectional	Male	100	On probation for IPV	32.2 (10.3)	MCTS	AAS	Avoidance (*r* = 0.28)
Karakurt et al., 2013	USA	Cross-sectional	Male	87	Couples of college students	22.3 (4.8)	CTS2	ECR; RQ	Attachment insecurity (*r* = 0.398 for M; *r* = 0.309 for F)
			Female	87					
Owens et al., 2014	USA	Cross-sectional	Male	133	Veterans in treatment for PTSD	51.28 (12.05)	CTS	ECR-S	Anxiety (*r* = 0.29) Avoidance (*r* = 0.30)
McKeown, 2014	UK	Cross-sectional	Female	92	Convicted	(NA)	CTS2	ECR-R	NSO
Brassard et al., 2014	Canada	Cross-sectional	Male	302	In treatment for relationship difficulties	35 (10.9)	CTS2	ECR	Anxiety (*r* = 0.34) Avoidance (*r* = 0.15)
Burk and Seiffge-Krenke, 2015	Germany	Cross-sectional	Male	194	High school students	16.99 (1.26)	CADRI	LEQ	Anxiety (*r* = 0.35 for M; *r* = 0.27 for F) Avoidance (*r* = 0.47 for M; *r* = 0.19 for F)
			Female	194		18.41 (2.02)			
Wright, 2015	USA	Longitudinal	Male	274	High school students	17.53 (0.51)	Cyber Aggression Self-Report	Attachment Self-Report	Anxiety (*r* = 0.33) Avoidance (*r* = 0.19)
Rodriguez et al., 2015	USA	Cross-sectional	Male	39	College students	22.51 (4.79)	CTS2	ECR	Anxiety (*r* = 0.35)
			Female	222					
Tougas et al., 2016	Canada	Cross-sectional	Male	210	Couples	43 (NA)	CTS2	ECR	NSO
			Female	210		41 (NA)			
Barbaro et al., 2016	USA	Cross-sectional	Male	258	Community	32.1 (8.9)	MRI-S	ECR	Anxiety (*r* = 0.66 for M; *r* = 0.66 for F) Avoidance (*r* = 0.29 for M)
Sommer et al., 2017	USA	Cross-sectional	Male	163	Couples	31.9 (9.51)	CTS2	AAS	Anxiety (*r* = 0.207 for M) Avoidance (*r* = 0.264 for M; *r* = 0.259 for F)
			Female	163		30.29 (9.61)			
Gou and Woodin, 2017	USA	Longitudinal	Male	69	Pregnant couples	34.71 (5)	CTS2	ECR	Anxiety (*r* = 0.30 for M)
			Female	71		32.24 (4.78)			
Cascardi et al., 2017	USA	Cross-sectional	Male	185	College students	19.5 (NA)	CADRI	RSQ	Anxiety (*r* = 0.33)
			Female	327					
Wright, 2017	USA	Cross-sectional	Male	276	College students	20.68 (0.61)	IPV Self-report	ECR-R	Anxiety (*r* = 0.36) Avoidance (*r* = 0.20)
			Female	324					

*IPV, Intimate Partner Violence; PMWI, Psychological Maltreatment of Women Inventory; RSQ, Relationship Style Questionnaire; RQ, Relationship Questionnaire; NA, Not available; CIR, Conflict in Relationships scale; MWA, Measure of Wife Abuse; ASQ, Attachment Style Questionnaire; PMP, Psychological Maltreatment of Partner; ECR, Experiences in Close Relationships Scale; HAI, History of Attachments Interview; CBI, Controlling Behavior Index; M, male; F, female; CTS2, Conflict Tactics Scale - Revised; CTS, Conflict Tactics Scale; NSO, Non-significant outcome; AAS, Adult Attachment Style; MMEA, Multidimensional Measure of Emotional Abuse; ECR-R, Experiences in Close Relationships Scale-Revised; DS, Dominance Scale; NVAWS, National Violence Against Women Survey modified to assess perpetration; ABI, Abusive behavior inventory; SSDS, Spouse-specific dependency scale; PMI, gender neutral version of Psychological Maltreatment of Women Inventory; IJS, Intimate Justice Scale; MIMARA, Multi-Item Measure of Adult Romantic Attachment; MCTS, Modified Conflict Tactics Scale; ECR-S, Experiences in Close Relationships Scale-Short form; CADRI, Conflict in Adolescence Rating Scale; LEQ, Love Experiences Questionnaire; MRI-S: Mate Retention Inventory-Short Form*.

Most of the studies have been conducted in America (64.28% in USA and 26.19% in Canada), with only two studies conducted in UK, one in Germany and one in Australia.

Studies have predominantly a cross-sectional design; only three studies present a longitudinal design (Lawson and Brossart, 2009; Wright, 2015; Gou and Woodin, 2017).

Surprisingly, only 22 studies investigated the topic among samples balanced for gender; instead, 16 studies had an exclusively male sample and 4 studies had an exclusively female sample.

Concerning sample nature, a relevant number of researches enrolled participants from community samples: most of them were conducted among high school and college students, couples and veterans in treatment for PTSD. Only 11 studies were focused on subjects with a history of IPV (Dutton et al., 1994, 1996; Dutton, 1995; Rankin et al., 2000; Mahalik et al., 2005; Mauricio et al., 2007; Lawson, 2008; Lawson and Brossart, 2009; Brown et al., 2010; Lawson and Malnar, 2011; McKeown, 2014).

Most of the studies were conducted among adult and young adult population, whereas only four studies investigated the topic among adolescents. According to the age of the samples, there was a consistent homogeneity over the instruments used to measure attachment: the most used instrument in adult sample was the ECR, both in its revised and its short version, although several studies used other measures.

Instead, concerning instruments to measure IPV, there was a remarkable variability presumably imputable to construct complexity, as psychological violence comprises very different forms of violence from verbal abuse to cyber aggression. It follows that the most used tool has been CTS, both in its revised and its short version, because of its ability to detect different types of psychological violence, less or more severe. Another common measure is the Psychological Maltreatment of Women Inventory (PMWI, Tolman, 1999), made up of several abuse typologies subscales. Other studies measured specific forms of violence with relevant instruments: as Controlling Behavior Index (BCI, Dobash et al., 1996), Dominance Scale (DS, Hamby, 1996), Intimate Justice Scale (IJS, Jory, 2004) and Mate Retention Inventory (MRI, Buss et al., 2008). In a longitudinal study conducted among high school students (Wright, 2015) there have been developed two specific self-report questionnaires to measure both attachment and cyber aggression.

Even though studies present a relevant heterogeneity, mainly for kind of violence and for sample composition, they supported the hypothesis of a relationship between insecure attachment and psychological IPV perpetration.

Four studies that confronted clinical groups of IPV perpetrators with control groups of non-perpetrators showed that attachment anxiety or fearful attachment make the difference between the two groups (Dutton et al., 1994, 1996; Dutton, 1995) and that IPV has a negative correlation (*r* = −0.30) with the attachment dimension of closeness (Lawson, 2008).

Concerning gender difference, there are contrasting results: even though most studies state that both male and female perpetrators tend to present attachment anxiety, some studies have found a prevalence of avoidant attachment in male samples compared to female (Lafontaine and Lussier, 2005; Gormley and Lopez, 2010; Frey et al., 2011; McKeown, 2014; Sommer et al., 2017).

The majority of studies support a positive correlation between psychological violence perpetration and the attachment dimension of anxiety, albeit the association indicated by coefficients ranged from 0.66 to 0.16. Regarding the association between avoidance and psychological IPV, several studies support the positive correlation between the two constructs, but the coefficient tend to be weak also in this case, ranging from 0.65 to 0.15.

#### Sexual IPV and attachment

As shown in Table 8, only seven studies investigated the relationship between sexual IPV and attachment from the perspective of the perpetrator.

**Table 8:** Studies investigating the relationship between sexual IPV and attachment among perpetrators.

**References**	**Country**	**Design**	**Sample characteristics**				**Instrument used to evaluate IPV**	**Instrument used to evaluate attachment**	**Main results**
			**Gender composition**	**Size**	**Type**	**Age**			
Kalichman et al., 1994	USA	Cross-sectional	Male	123	College students	19.6 (1.33)	CTS	Attachment Security Ratings	NSO
Rankin et al., 2000	USA	Cross-sectional	Male	69	Convicted for IPV	31 (11)	MWA	ASQ	Avoidance (*r* = 0.29)
Smallbone and Dadds, 2001	Australia	Cross-sectional	Male	119	College students	21 (5.41)	SES	ECR	Anxiety (*r* = 0.19) Avoidance (*r* = 0.28)
Ménard et al., 2010	USA	Cross-sectional	Male	148	College students	20.3 (2.2)	SEQ	ASQ	Anxiety (*r* = 0.25) Avoidance (*r* = 0.22)
			Female	278					
Karakurt et al., 2013	USA	Cross-sectional	Male	87	Couples of college students	22.3 (4.8)	CTS2	ECR	NSO
			Female	87					
He and Tsang, 2014	China	Cross-sectional	Male	439	College students	20.87 (1.45)	SCIRS	ECR-S	Anxiety (*r* = 0.32)
			Female	488					
Sommer et al., 2017	USA	Cross-sectional	Male	163	Couples	31.9 (9.51)	CTS2	AAS	Anxiety (*r* = 0.24 for M; *r* = 0.16 for F) Avoidance (*r* = 0.22 for M)
			Female	163		30.29 (9.61)			

*CTS, Conflict Tactics Scale; IPV, Intimate Partner Violence; MWA, Measure of Wife Abuse; ASQ, Attachment Style Questionnaire; SES, Sexual Experiences Survey; ECR, Experiences in Close Relationships Scale; SEQ, Sexual Experiences Questionnaire; CTS2, Conflict Tactics Scale-Revised; NSO, Non-significant outcome; SCIRS, Sexual Coercion in Intimate Relationships Scale; ECR-S, Experiences in Close Relationships Scale-Short form; AAS, Adult Attachment Style; M, male; F, female*.

The majority of these studies have been conducted in USA (71.42%), one study has been run in Australia and another one in China. No studies have yet been conducted in Europe. All the studies present a cross-sectional design.

Although sexual partner violence is usually considered a male preserve, most researches explored the construct among samples balanced for gender and only three studies present a male-only sample (Kalichman et al., 1994; Rankin et al., 2000; Smallbone and Dadds, 2001).

Regarding sample composition, most of the studies have been conducted among community population samples, like college students (Kalichman et al., 1994; Smallbone and Dadds, 2001; Ménard et al., 2010; Karakurt et al., 2013; He and Tsang, 2014) and community couples (Sommer et al., 2017). Surprisingly, only one study has been focused on male subjects convicted for IPV (Rankin et al., 2000).

All the studies were conducted among young adult and adult population, so all the samples are relatively homogeneous for age; due to the peculiarity of the construct, it has not been investigated among adolescents.

Even though the populations of different samples are not homogeneous, there is a consistent heterogeneity in the choice of instruments, both for IPV and for attachment measures. Concerning attachment measures, ECR, both in its standard and its short version and Attachment Style Questionnaire (ASQ, Feeney and Noller, 2001) are the most common instruments. There is much more variability regarding the measures that assess sexual IPV: only three studies made use of CTS, in its original and in its revised version, which is the most widespread instrument to assess different forms of IPV. Some studies employed very specific measures that consider sexual experiences more broadly, among which we find sexual coercion and sexual abuse perpetration.

All studies, except two that did not have any significant outcome, support the hypothesis of a positive correlation between attachment dimensions and sexual IPV perpetration. An equal number of studies support the correlation between sexual IPV and the attachment dimension of anxiety and the correlation between sexual IPV and the attachment dimension of avoidance. Concerning the dimension of anxiety, several studies supported the correlation with sexual IPV (Smallbone and Dadds, 2001; Ménard et al., 2010; He and Tsang, 2014; Sommer et al., 2017). The coefficients presented ranged from 0.32 to 0.16, that indicate a weak correlation. The positive correlation between the attachment dimension of avoidance and sexual IPV is supported by several studies, but the coefficient is not strong, as for anxiety, ranging from 0.29 to 0.22.

Regarding gender difference, one study (Sommer et al., 2017) confronted male and female groups and it resulted that both female and male sample presented a significant rate of attachment anxiety but only male results displayed a correlation between sexual IPV and avoidance.

### New lines of research

In the studies we examined, there were several studies that studied the construct of IPV and its relationship with attachment in peculiar groups or from an unconventional perspective, which could represent interesting future trends in research.

Some studies investigated IPV in homosexual individuals and couples, to see if they are consistent with results obtained in heterosexual couples. Other studies were focused on couples and mutual violence, which are a privileged point of view to investigate how the phenomenon of IPV arise in dyads, considering couple attachment. In the end, there is a specific current of studies that explored the chance to categorize IPV perpetrators in specific groups, to develop specific approaches to treatment that take into account the peculiar characteristics of the specific groups.

#### IPV in same-sex couples

As Table 9 illustrates, five studies (Landolt and Dutton, 1997; Stanley et al., 2006; Bartholomew et al., 2008; Craft et al., 2008; Gabbay and Lafontaine, 2017), in a time lapse that runs from 1997 to 2017, investigated the relationships between different forms of IPV among same-sex couples and homosexual individuals. These studies have been conducted only in Canada (60%) and USA (40%), maybe due to the major cultural acceptance of homosexuality in these Countries. All the studies, except one, investigated the correlations between the two constructs in community samples, mainly recruited by advertising on newspaper and on the internet. All the studies have been conceived with a longitudinal design.

**Table 9:** Studies investigating the relationship between generic IPV and attachment among homosexuals.

**References**	**Country**	**Design**	**Sample characteristics**				**Instrument used to evaluate IPV**	**Instrument used to evaluate attachment**
			**Gender composition**	**Size**	**Type**	**Age**		
Landolt and Dutton, 1997	USA	Longitudinal	Male couples	52	Community	34 (NA)	PMI; CTS	RSQ
Stanley et al., 2006	Canada	Longitudinal	Male	69	Experienced IPV	38.6 (8.2)	IPV Interview	History of Attachments Interview
Bartholomew et al., 2008	Canada	Longitudinal	Male	186	Community	38.53 (9.44)	CTS	RSQ
Craft et al., 2008	USA	Longitudinal	Male	46	Community	33.52 (8.97)	CTS2	RSQ
			Female	41		30.20 (9.38)		
Gabbay and Lafontaine, 2017	Canada	Longitudinal	Male	107	Community	46.88 (12.46)	CTS2	ECR
			Female	203		43.19 (11.17)		

*NA, Not available; PMI, Psychological Maltreatment Inventory; CTS, Conflict Tatics Scale; RSQ, Relationship Style Questionnaire; IPV, Intimate partner Violence; HAI, History of Attachments Interview; CTS2, Conflict Tactics Scale-Revised; ECR, Experiences in close relationship*.

Concerning the instruments, the majority of studies made use of CTS, both in its original and in its revised version; one study used a specific interview about history of IPV experiences and another one used both CTS and Psychological Maltreatment Inventory (PMI) to assess both physical and psychological violence. Regarding attachment, the most used instrument is the Relationship Scale Questionnaire (RSQ, Bartholomew and Horowitz, 1991).

Concerning the composition of samples, only two studies assessed samples with a gender balanced composition; two studies were conducted among male populations; one study, instead, enrolled male couples.

All the studies confirm the positive correlation among homosexual population between different typologies of IPV and attachment, both for perpetrators and victims, in accordance with the findings obtained in researches that investigated the subject in heterosexual population.

Among all the studies, only two investigated the constructs from both the perspectives; the others were focused on perpetrators.

According to Bartholomew et al. (2008), IPV victims in same sex relationships show a negative correlation between avoidance and both psychological (−0.17) and physical (−0.16) IPV, whereas there was a positive correlation between anxiety and physical IPV (*r* = 0.17). For what concerns IPV perpetrators in same sex relationships, the study claimed a positive correlation between anxiety and both psychological (*r* = 0.18) and physical (*r* = 0.19) IPV and a negative correlation between avoidance and physical IPV (*r* = −0.22).

Concerning studies that assessed the construct only among perpetrators, there is a general accordance about the positive correlation between insecure attachment and generic IPV perpetration (Craft et al., 2008). Concerning psychological IPV (Landolt and Dutton, 1997; Bartholomew et al., 2008), perpetration turned out to be positively correlated to the attachment dimension of anxiety (*r* = 0.18) and to fearful (*r* = 0.40) and preoccupied (*r* = 0.26) and negatively correlated to secure attachment styles (*r* = −0.37). For physical IPV (Landolt and Dutton, 1997; Bartholomew et al., 2008), correlations are different: perpetration of this form of violence is positively correlated with fearful attachment style (*r* = 0.34) and with the attachment dimensions of anxiety (*r* = 0.19), but it is negatively correlated with avoidance (*r* = −0.22). Only one study investigated the relationship between sexual IPV perpetration and attachment and both the attachment dimensions of anxiety and avoidance are positively correlated with the construct.

The study that assessed the relationship between IPV and attachment using History of Attachments Interview (Stanley et al., 2006) deserve a particular mention: due to the peculiar structure of the interview, that explores experiences concerning significant attachment episodes along lifespan, several significant themes recurred in people narratives. Unmet or threatened emotion needs, such as need for closeness or desire for commitment and monogamy or loss of relationships, recurred as consistent themes in participant's stories. So the authors agreed that they can be interpreted as attachment wounds linked to dimensions of anxiety and avoidance.

#### IPV and attachment in couple contexts

To date, 15 studies investigated the relationship between IPV and attachment in couples, taking in consideration both partners attachment (see Table 10).

**Table 10:** Studies investigating the relationship between IPV and attachment in couple context.

**References**	**Country**	**Design**	**Sample characteristics**				**Instrument used to evaluate IPV**	**Instrument used to evaluate attachment**
			**Gender composition**	**Size**	**Type**	**Age**		
Bookwala, 2002	USA	Cross-sectional	Male	59	College students	19 (NA)	CTS	RSQ
			Female	102				
Bond and Bond, 2004	Canada	Cross-sectional	Male	43	Community couples	41.83 (11.48)	PAS-P; PAPS; MSI-R	RQ; ECR
			Female	43		39.85 (10.26)		
Rogers et al., 2005	USA	Cross-sectional	Male	80	College couples	20.71 (3.66)	CTS	AAQ
			Female	80		19.54 (3.4)		
Allison et al., 2008	Canada	Cross-sectional	Male	23	Couples in treatment for IPV	34.13 (8.18)	IPV Interviews	HAI
			Female	23		33.7 (9.39)		
Doumas et al., 2008	USA	Cross-sectional	Male	70	Community couples	28.46 (10.36)	CTS	RQ
			Female	70		7.03 (10.52)		
Rapoza and Baker, 2008	USA	Cross-sectional	Male	171	Community couples	19.77 (3.06)	CTS2	Attachment Security Ratings
			Female	171				
Miga et al., 2010	USA	Longitudinal	Male	93	Community adolescents	14.28 (0.78)	CIR	ECR
Frey et al., 2011	USA	Cross-sectional	Male	20	Couples in treatment for PTSD	28.5 (5.11)	IJS	MIMARA
			Female	20		28.2 (6.24)		
Kuijpers et al., 2012	Netherlands	Longitudinal	Female	74	IPV victims	39.28 (10.04)	CTS2	ECR-S
Péloquin et al., 2011	Canada	Cross-sectional	Male	193	Community couples	31 (NA)	CTS2	ECR
			Female	193				
Wilson et al., 2013	USA	Cross-sectional	Male	696	Community couples	43 (NA)	IPV self-report	ECR
			Female	696				
Bélanger et al., 2015	Canada	Cross-sectional	Male	20	Couples in treatment for IPV	34.3 (NA)	CTS2	ECR-S
			Female	20		32.2 (NA)		
Bookwala and Zdaniuk, 1998	USA	Cross-sectional	Male	26	College students	19 (NA)	CTS	RQ
			Female	59				
Seiffge-Krenke and Burk, 2015	Germany	Cross-sectional	Male	194	Couples of high school students	16.99 (1.26)	CADRI	ECR
			Female	194		18.41 (2.02)		
Goncy and van Dulmen, 2016	USA	Cross-sectional	Male	113	Couples of college students	20.25 (1.8)	CADRI	ECR-R
			Female	113		19.13 (0.8)		
Smith and Stover, 2016	USA	Longitudinal	Female	93	IPV victims	30 (NA)	CTS2	ECR-R
Lewis et al., 2017	USA	Cross-sectional	Male	296	Pregnant adolescent couples	21.3 (4.1)	CTS	ECR
			Female	296		18.7 (1.7)		

*IP, Intimate Partner Violence; NA, Not available; CTS, Control Tactics Scale; RSQ, Relationship Style Questionnaire PAS-P, Partner Abuse Scale-Physical; PAPS, Physical Abuse of Partner Scale; MSI-R, Marital Satisfaction Inventory-Revised; RQ, Relationship Questionnaire; ECR, Experiences in Close Relationship; AAQ, Adult Attachment Questionnaire; HAI, History of Attachments Interview; CTS2, Conflict Tactics Scale; CIR, Conflict in Relationships; IJS, Intimate Justice Scale; MIMARA, Multi-Item Measure of Adult Romantic Attachment; ECR-S, Experiences in Close Relationships Scale-Short form; CADRI, Conflict in Adolescence Rating Scale*.

Most of the studies were run in North America, in USA (66.6%) and Canada (33.3%), and only one study was conducted in Europe (Germany). All the studies, except two (Bookwala and Zdaniuk, 1998; Miga et al., 2010), were conducted among couple samples and only one study investigated the construct among a sample not balanced for gender (Miga et al., 2010). Concerning the design of the studies, all adopted a cross-sectional design except one (Miga et al., 2010).

There was an interesting variability concerning sample composition: some studies investigated the construct among community samples (Bond and Bond, 2004; Doumas et al., 2008; Rapoza and Baker, 2008; Péloquin et al., 2011; Wilson et al., 2013), some among college students (Bookwala and Zdaniuk, 1998; Bookwala, 2002; Rogers et al., 2005; Goncy and van Dulmen, 2016), three studies among adolescents (Miga et al., 2010; Seiffge-Krenke and Burk, 2015; Lewis et al., 2017) and one study among veterans in treatment for PTSD and their spouses (Frey et al., 2011).

Surprisingly, only two studies regarded couples presenting previous or current IPV (Allison et al., 2008; Bélanger et al., 2015).

Due to the heterogeneity of the samples, there was a remarkable variability of instruments used. Concerning attachment, the most common instrument was ECR, also in its revised and in its short version, but other instruments have been used. As for attachment, a wide range of instruments has been used also to assess IPV: CTS, also in its revised version, has been used in the majority of researches.

Even though the studies adopted different instruments to measure the construct and different methods to test the hypothesis, all of them agree on the positive correlation between IPV and insecure attachment of both partners. According to studies that investigated the relationship between partners attachment and IPV, not making distinctions between different forms of violence, mutual violence seems to occur when there is at least on partner with preoccupied attachment (Bookwala and Zdaniuk, 1998; Bookwala, 2002), in the dismissing-anxious couple pattern (Bond and Bond, 2004), when both partners present anxious attachment (Allison et al., 2008), when they show high rates on both attachment dimensions of anxiety and avoidance (Rogers et al., 2005).

The dismissing-anxious pattern seems to be the most common configuration in couples with one-sided IPV (Bookwala, 2002; Frey et al., 2011; Wilson et al., 2013). This pattern of interaction has been referred to as a pursue-withdraw cycle (Johnson, 2004). But several studies claim that one sided IPV couples have at least one partner showing high scores on the attachment dimension of anxiety (Lewis et al., 2017).

Concerning physical IPV and partner's attachment, the dismissing-anxious couple pattern seems to foster physical violence in the couple (Rogers et al., 2005; Doumas et al., 2008), even in adolescents (Miga et al., 2010). One study, conducted by Bélanger et al. (2015), reported a positive correlation between physical IPV victimization and avoidant attachment in males.

Other studies claimed that partner presenting high rates of anxiety tend to foster physical IPV perpetration by the other partner (Doumas et al., 2008; Rapoza and Baker, 2008; Seiffge-Krenke and Burk, 2015) or even by themselves (Péloquin et al., 2011).

Concerning the relationship between psychological IPV and attachment in couples, some studies claim that high levels of attachment anxiety in one of the members of the couple foster IPV perpetration by the other (Péloquin et al., 2011; Goncy and van Dulmen, 2016), while other studies assert that high levels of both anxiety and avoidance by one of the partners are positively related to psychological IPV (Seiffge-Krenke and Burk, 2015).

Two interesting studies that tried to explain the complex mechanism of mutual IPV in couples have been conducted in Netherland (Kuijpers et al., 2012) and in USA (Smith and Stover, 2016). These longitudinal studies accurately investigated the phenomenon of IPV revictimization and use of violence by IPV victims among samples of IPV victims although they got contrasting findings. In accordance with Kuijpers et al. (2012) avoidant attachment is a significant predictor for revictimization of both psychological and physical IPV; conversely, Smith and Stover (2016) found a positive correlation with anxious attachment in victims and IPV revictimization and use of violence.

#### IPV profiles and attachment among perpetrators

Between 1998 and 2014, eight studies investigated the relationship between IPV perpetration and attachment, identifying different typologies of perpetrators. Characteristics of these studies are showed in Table 11. The studies were conducted in USA (75%) and in Netherlands (25%) and they all adopted a cross-sectional study design.

**Table 11:** Studies investigating the relationship between IPV profiles and attachment among perpetrators.

**References**	**Country**	**Design**	**Sample Characteristics**				**Instrument used to evaluate IPV**	**Instruments used to evaluate attachment**
			**Gender composition**	**Size**	**Type**	**Age**		
Tweed and Button, 1998	USA	Cross-sectional	Male	79	In treatment for IPV	35	CTS	RSQ
Holtzworth-Munroe et al., 2000	USA	Cross-sectional	Male	102	Violent	35.62 (9.26)	CTS2	RSQ
				62	Non-violent			
Waltz et al., 2000	USA	Cross-sectional	Male	75	Violent	34.17 (NA)	CTS	AAS
				32	Non-violent	42.31 (9.82)		
Mauricio and Gormley, 2001	USA	Cross-sectional	Male	60	In treatment for IPV	29 (NA)	CTS	RQ
Mauricio and Lopez, 2009	USA	Cross-sectional	Male	304	In treatment for IPV	33 (8.99)	CTS	ECR
Chiffriller and Hennessy, 2009	USA	Cross-sectional	Male	201	In treatment for IPV	35.10 (10.16)	CTS2	RSQ
Buck et al., 2012	Netherlands	Cross-sectional	Male	72	In treatment for IPV	35.5 (7.98)	CTS2	RQ
				62	Non-violent	39.5 (10.1)		
Buck et al., 2014	Netherlands	Cross-sectional	Male	72	In treatment for IPV	Sec: 34.9 (7.9) Insec: 35.8 (8.1)	CTS2	ECR
				62	Non-violent	Sec: 36.4 (9.2) Insec: 45 (11.8)		

*IPV, Intimate Partner Violence; CTS, Conflict Tactics Scale; RSQ, Relationship Style Questionnaire; NA, Not available; AAS, Adult Attachment Style; RQ, Relationship Questionnaire; CTS2, Conflict Tactics Scale - Revised; ECR-R, Experiences in Close Relationship - Revised; ECR, Experiences in Close Relationship*.

All the studies enrolled male participants. Seven of them were conducted among clinical populations of people in treatment for IPV, only two studies investigated the construct among a community sample, recruited by advertising (Holtzworth-Munroe et al., 2000; Waltz et al., 2000).

Concerning instruments, there was a surprising homogeneity about the instruments used to assess violence: all the researches make use of CTS, both in its original and in its revised version. To evaluate the construct of attachment, several instruments have been chosen: the most common instruments is ECR, both in its original and in its revised version (ECR-R).

Concerning classifications of perpetrators made by the studies, there are several interesting differences. Three studies distinguished perpetrators according to attachment style (Mauricio and Gormley, 2001; Buck et al., 2012, 2014); four other studies according to violence level or typology (Holtzworth-Munroe et al., 2000; Waltz et al., 2000; Chiffriller and Hennessy, 2009; Mauricio and Lopez, 2009); one study identified specific categories of perpetrators, based on several characteristics (Tweed and Button, 1998).

Considering classifications based on attachment styles, Mauricio and Gormley (2001) spotted two categories among violent men in treatment for IPV: Insecurely attached and Securely attached. At a primary analysis, men with secure attachment showed a higher level of social desirability and a lower need for dominance, but apparently the same violence level. Controlling for social desirability, men reporting insecure attachment showed significantly higher scores on CTS than men reporting secure attachment.

According to the study conducted by Buck et al. (2012), who also divided perpetrators in securely and insecurely attached, men that reported attachment insecurity showed higher separation anxiety, higher distrust in partner, higher dependency, lower self-esteem and more impulsivity. They hypothesized that distrust and separation anxiety might explain insecurely attached men proneness to commit IPV.

The following study conducted by Buck et al. (2014) used a different measure to assess attachment, ECR and distinguished even controls in two group according to attachment security. It resulted that securely attached perpetrators do not differ, concerning attachment scores, from securely attached control group; also insecurely attached batterers and insecurely attached controls presented the same attachment scores. So attachment seems to act as a mediator between personality disorder traits and committing violence toward the partner.

Concerning groups distinguished according to violence, Holtzworth-Munroe and Stuart, in 1994, hypothesized a classification based on severity of marital violence, generality of violence and psychopathology or personality disorders. After analyzing previous literature on the subject, they grouped IPV perpetrators in three categories: Family-only batterers (less severe marital violence, lowest level of general violence, lowest psychopathology), Borderline-dysphoric batterers (moderate-high severity of marital violence, moderate level of general violence, moderate-high psychopathology) and Generally violent-antisocial batterers (very severe marital violence, highest level of general violence, highest psychopathology). Concerning romantic attachment, according to their findings, Family-only batterers tend to have a prevalence of secure or preoccupied attachment, Borderline-dysphoric batterers a prevalence of preoccupied attachment, whereas Generally violent-antisocial batterers a prevalence of Dismissing attachment.

The same team of researchers (Holtzworth-Munroe et al., 2000), conducted a research to test their categorization several years later and added a fourth category: Low level antisocial that presented, compared to Family-only category, more severity in marital violence, a higher level of general violence and same psychopathology levels. The results are consistent with the hypothesis in the previous study (Holtzworth-Munroe and Stuart, 1994) concerning attachment, even though severity of both couple and general violence and level of psychopathology are lower, having been tested on a community sample.

Along with these findings, Waltz et al. (2000) identified three groups, very similar to the classification conceived by Holtzworth-Munroe and Stuart (1994): Generally violent men, Pathological violent men and Family only violent men. Men belonging to the first group showed higher scores on attachment dimension of avoidance and lower scores on anxiety, while Pathological violent men showed lower attachment avoidance scores and higher scores on anxiety. Family only batterers, instead, showed attachment anxiety and avoidance scores similar to control group.

In the research conducted by Mauricio and Lopez (2009), the sample has been divided into three categories based on violence level: Low level violence, Moderate level violence, High level violence. It turned out that men belonging to the Low level violence group reported more acts of physical violence (even though it was about non-severe acts) than other groups, even though they presented attachment rates similar to community samples. Men belonging to the Moderate level violence group reported higher scores on the attachment dimension of anxiety; while men belonging to the High level violence group reported high levels on both the dimensions of anxiety and avoidance.

In the study conducted by Chiffriller and Hennessy (2009), the sample of IPV perpetrators was divided in five categories, three of which have been considered in the research conducted by Waltz et al. (2000). Chiffriller and Hennessy (2009) identified two more categories, new in literature, to fit Sexually violent and Psychologically violent perpetrators. Concerning attachment, men belonging to the Pathological violent category resulted to be more preoccupied (low avoidance and high anxiety) and more fearful (high avoidance and high anxiety) than men belonging to other groups. So results are partially concurring with those found by Waltz et al. (2000).

Tweed and Button (1998) made a peculiar classification of IPV perpetrators identifying two categories: Instrumental batterers and Impulsive batterers, based on participant's personality characteristics. So one group included generally violent/antisocial men and the other was made up of men that presented dysphoric/borderline cluster characteristics. Instrumental batterers got higher scores on preoccupied attachment, while Impulsive batterers presented a less secure and more fearful attachment.

## Discussion

The aim of this paper was to review empirical studies investigating the relationship between attachment and IPV and going through two main research questions: Is attachment involved in IPV? How attachment may explain the processes leading to IPV?

### Main findings

#### Characteristics of the studies

Studies often adopted a cross-sectional design of research and were more rarely longitudinal. The main and common hypothesis underlying research was the conceptualization of IPV as an outcome determined by the quality of attachment. This hypothesis makes sense when referring to IWM as developing within the very early interpersonal context of an individual and maintaining a stability across life-span (Bowlby, 1988). In this perspective, the investigation of IWM may be recommended for studies examining variables related to childhood maltreatment or past life trauma. However, few studies used AAI to evaluate the quality of attachment in relation to IPV with the most of the them measuring romantic attachment related to a current relationship. Despite the fact that romantic adult attachment styles are thought to mainly depend on early internal working models, it is not the case for all individuals (Fraley, 2002). This approach seems reasonable when the investigators are interested in dyadic variables (e.g., marital satisfaction or conflict levels). But it could be also argued that the quality of romantic attachment is determined by IPV, reversing the initial hypothesis. Indeed, this has been successfully tested in one of the study reviewed in our paper. As a whole, future studies investigating the relationships between the quality of attachment to parents and IPV should prefer longitudinal design of research or the use of valid instrument.

Another central issue is related to the instruments used across the whole literature. Indeed, the prevalent use of self-report questionnaire and the poor (or inexistent) use of interview (e.g., AAI or CRI) did not allow to evaluate sufficiently the role of disorganized attachment in IPV. This issue could be especially problematic because of the role of past life trauma in IPV. Trauma in early childhood has been showed to be often associated with a disorganized quality of attachment, which in turn is associated with a wide range of negative outcomes as such as personality disorders and violence (Rholes et al., 2016). This important gap has to be fulfilled by future research which should select congruent instruments to estimate the role of disorganized attachment in IPV victimization and perpetration.

#### Focus of the studies: forms of violence

Most of the study reviewed in this paper focused on physical and psychological violence and neglected the topic of sexual IPV. Noteworthy, sexuality is considered as the most exclusive domain of romantic relationship and is thought to be tightly related to the development of attachment model (Lichtenberg et al., 2007). In that sense, sexual violence perpetrated by a romantic partner is perhaps the most intimate form of IPV. Interestingly, sexual IPV has been often included in studies examining the relationship between attachment and IPV without differentiating between different forms of violence. As recently stated, research toward sexual IPV is plagued by important difficulties related to the definition of the construct and the lack of instruments available for its assessment (Bagwell-Gray et al., 2015). The few results reviewed in our paper are somewhat contrasting. Despite most of the studies found an association between insecure attachment and sexual IPV victimization, only one research examined the topic among participants with a previous reported history of IPV victimization (Smagur et al., 2018). In relation to sexual IPV perpetration, studies showed that perpetrators were not only anxious (as for the other types of violence) but also avoidant. These preliminary data are in line with the subtyping model of batterers proposed by Holtzworth-Munroe and Stuart (1994). The authors asserted that perpetrators of sexual IPV belong to the most severe group of batterers, often showing antisocial personality traits. However, further studies investigating the role of attachment in sexual IPV should be conduct to increase current understanding of the topic and to offer indications for the tailoring of treatment programs.

Also, another fundamental issue has been neglected by the studies examining the relationship between attachment and IPV: the topic of polivictimization. Indeed, researchers generally decided to create a non-specific index of IPV or to differentiate between several forms of violence. However, studies did not examined differences between single-form or combined-form IPV experiences. As polivictimization has been showed to be associated with worse outcomes, such gap should be fulfilled by future researches following the lines of Ross et al. (2016).

#### IPV and anxious attachment

Anxious attachment has been early thought to act as a risk factor for IPV victimization. Indeed, an individual with anxious attachment is usually described as suffering from fear of abandonment and high levels of separation anxiety. They may have difficulty to leave abusive relationships because the loss of the partner is experienced as unbearable and generate so much anxiety that the individual may prefer the least worse option. Similarly, the fact that anxious individuals suffer from low self-esteem (Mikulincer and Shaver, 2005) may lead them to think to not have sufficient resources to front a separation by the abusive partner. These individuals may be especially prone to deceive themselves about the possibility that partner will change. Also, anxious attachment is usually related to a negative self-image, as underserving of love and care. Together with low self-esteem, these characteristics may lead to self-attribution, in terms of responsibility, of IPV. Importantly, a violent partner who is intermittently loving and attending may further reinforce this interpersonal pattern, increasing the value of the relationship the individual fears to loss and favoring illusions about a future change of partner behavior. Unfortunately, these considerations about the mechanisms that operate in the link between anxious attachment and IPV victimization remain speculative, as too few studies included in their research design an examination of other potential variables as for example perception of social support or self-esteem.

In relation to IPV perpetration, results suggest a convergence in literature showing that batterers are prone to be anxiously attached to their partners. Importantly, these findings have been found in relation to every form of IPV. This is in line with the attachment theory of IPV, which assert that an anxious individuals tend to hyperactivate the attachment system, exaggerating protestation signals when attachment needs are not met. For example, they are especially demanding in terms of caregiving and love demonstrations. Also, when attachment needs are not met, they tend to use extreme forms of emotion regulation strategies that generally involve the interpersonal domain (e.g., being reassured by the partner). This hypothesis seems further supported by studies showing the role played by high levels of anger in anxiously attached perpetrators. Violence has been identified as one of these regulation strategies that anxious individuals may use when feeling too much frustration. This consideration may explain results identifying a high prevalence of anxiously attached individual among a specific subtype of batterers, violent only in family relationships (Holtzworth-Munroe et al., 2000). Indeed, they would use violence only to obtain satisfaction of their attachment needs, in intimate context.

The anxious component of attachment has also been involved in the dyadic explanation of IPV. If both partners are anxiously attached, conflict resolution strategies would be probably dominated by engagement by both partners, leading to an escalation of conflict and terminating in episodes of violence (Bonache et al., 2017). Indeed, studies investigating the attachment matching in violent couples showed that couples where both partner have an anxious attachment are more prone to be violent (Bookwala and Zdaniuk, 1998; Bookwala, 2002). Also, anxious interpersonal regulation strategies may lead the partner to retreat from the relationship, further intensifying the frustration of attachment needs and potentially triggering an escalation of intimate conflict, terminating in violent acts. Noteworthy, this pattern could be especially true when one partner has an avoidant attachment, tending to withdraw from conflicts.

#### IPV and avoidant attachment

Turning to the avoidance dimension of attachment, our review showed that near half of the studies found a relationship between avoidant attachment and IPV victimization. At an individual level, some consideration can be made in an attempt to understand why an avoidant victim of IPV does not leave a violent relationship. For example, avoidant individuals have typical difficulties in seeking help because of some dysfunctional beliefs. They are generally convinced that showing personal difficulties and vulnerabilities to others is unbearable as they expect that help request would be reject by others, fundamentally unavailable. As such, lack of social support, tightly related to IPV victimization (Zapor et al., 2018), may be very high among avoidant victims of IPV (Davis et al., 2002). Also, avoidant individuals may underestimate the psychological costs of IPV violence, being erroneously convinced to be psychologically immune to emotional threats. Supporting this idea, most of the evidences supporting a link between IPV and avoidant attachment has been brought in relation to psychological IPV victimization.

Turning to the other side of IPV, some studies reviewed in this paper showed that perpetrators are often avoidant in their attachment styles. However, empirical evidences are contrasting. Importantly, models offering a sub-classification of batterers are insightful. Indeed, it has been asserted that a subtype of individuals, being mostly antisocial and highly violent, are especially prone to be avoidant (Waltz et al., 2000). For this subtype, violence may be used as a way to control and manipulate the partner, exerting a politics of fear. This is in line with results showing that gender role stress mediates the relationship between attachment insecurity and controlling behavior among male batterers (Mahalik et al., 2005).

Moreover, these individuals are not only physically aggressive but also use psychological and sexual violence. Interestingly, our review seems to support such hypothesis underlying that the proportion of study finding significant associations between avoidant attachment and IPV are higher in the sexual and psychological sections compared to the physical section. Again, it is highlighted the proficiency to examine the topic of IPV differentiating between forms of violence. Noteworthy, regarding sexual violence, it has been showed that avoidance was significantly associated with IPV only among male and not among female. Following the idea that sexual violence in avoidant individuals may be a way to control the partner, this result makes sense as gender differences in sexuality are often attributed to a different valence in terms of dominance motivation (Malamuth, 1998; Toates et al., 2017).

At a dyadic level, avoidant individuals may elicit in the partner high activation of the attachment system because of a tendency to withdraw and retreat from the relationship. From this perspective, it has been asserted that disengagement during conflicts may be interpreted in the light of an abandonment threat by anxious attached individuals. Such behaviors, in conflictual context, may exasperate the frustration of the anxiously attached partner who in turn would be more prone to use violence as an extreme form of protestation. For example, Bonache et al. (2017) found that among boys, avoidant attachment was related to IPV victimization through self-reported withdrawal strategies and conflict engagement behaviors attributed to their partner. However, there is still lack of studies testing such hypotheses. Future research should include in their investigation other variables, as for example pathological personality and measures of romantic relationship power and dominance.

#### The role of gender and sexual orientation

As the proportion of men and women being involved in IPV victimization and perpetration is highly unbalanced, it is not surprising that a numbers of researchers explored the role of gender in the association between IPV and attachment. For example, some studies found that the relationship between avoidant attachment and both physical (Bond and Bond, 2004; Karakurt et al., 2013) and sexual (Sommer et al., 2017) IPV victimization was significant only among males. However, some authors found an inverse pattern of results with associations between insecure attachment and IPV victimization being significant only among women (Péloquin et al., 2011; Hellemans et al., 2015). Recently, it has been argued that gender discrepancies may be due to the matching between gender and type of conflict resolution strategies used. Bonache et al. (2017) argued that conflict resolution strategies, which are not in line with gender expectations, might be less accepted and consequently elicit the use of violence by the other partner. In this regard, studies conducted on homosexual population may be a profitable perspective from which observe the role of gender in the relationship between attachment and IPV. For example, Bartholomew et al. (2008) found that avoidance was negatively related to IPV victimization and perpetration among their sample of homosexual men. This contrasts with the studies, illustrated above, conducted among heterosexual population of men suggest that gender expectancies may play a role in the relationship between avoidance and IPV victimization. However, too few studies explore the topic among samples of homosexual individuals and mainly neglected IPV victimization. Indeed, if preliminary results toward IPV perpetration converge with those obtained among heterosexual population, further research is needed to increase the understanding of the topic.

## Conclusions

Our paper aimed to provide a complete review of empirical evidences investigating the role of attachment in relation to IPV.

Importantly, a great number of studies failed to find significant associations between insecure attachment and IPV victimization or perpetration. However, preliminary results evidenced that victims and perpetrators of IPV are heterogeneous population in relation to attachment. Importantly, IPV is not a deterministic phenomenon and the complex and multidimensional relationships between an individual, her/his resources and the risk factors occurring at different steps of the relationship should be considered. Indeed, a possible explanation is that anxious, avoidant and secure individuals might be at risk of experience or perpetrate IPV but for different reasons. In line with this, the investigation of the relationships between attachment and others central associates of IPV may further shed light on such issue. However, the literature reviewed in the paper often neglected the role of other important correlates of IPV. For example, attachment and IPV have been rarely investigated in relation to poverty or among populations of minority women. Indeed, too few studies include in their design other variables that may interact with attachment styles and explain the heterogeneity of these results.

Some interesting clinical implications might be drawn from this examination. First, attachment theory asserts that IWM could change over time in the context of secure and supportive relationships. As such, increasing social support and reinforcing the development of secure romantic relationships should be encouraged by clinicians. Also, clinicians themselves might provide a secure base to both victims and perpetrators in order to alter insecure IWM and to shift toward a secure one. Also, when working with victims of IPV, clinicians might guide patients toward an increased awareness of how attachment issues have affected their relationship. For instance, anxious attachment may include an awareness of the value of the relationship. The clinician may support the patient to maintain such awareness, framing it in a more positive way. However, clinicians should avoid the reinforcement of the erroneous attribution of internal blames for IPV that anxious victims may show. Instead, this therapeutic process should be promoted by a clinical support in the development of coherent narratives of early attachment experiences. Then, some potential mediating variables explaining the relationship between insecure attachment and IPV victimization and perpetration might be the target of treatment. For instance, emotion regulation capacities (Garofalo and Velotti, 2015; Balzarotti et al., 2016) and especially deficit in the capacity to regulate anger (Garofalo and Velotti, 2017; Velotti et al., 2017) may be a strategic objective in the treatment of perpetrators. Finally, communication capacities related to attachment needs should be improved in both perpetrators and victims. For example, anxious perpetrators should be supported in their capacity to interpret disengagement from partner and to better tolerate and communicate emotions related to interpersonal rejection.

Despite the important contribution provided by this paper, some limitations should be pointed out. For instance, publication bias is a well-documented limitation of systematic reviews and the present work is not an exception. Indeed, we excluded not published studies potentially leading to a misrepresentation of findings in the field. Especially, studies with inconsistent or negative results are hard to publish. Also, most of the studies reviewed in our paper are cross sectional in their design, limiting the possibility to made causal inferences. Specifically, the inability to answer to the question of whether attachment styles precede IPV and to what extent they are the result of psychological changes and specifically changes in interpersonal area, as consequences of IPV, remains a central issue. Finally, in our systematic and qualitative review, we included all studies without important exclusion criteria regarding their methodological quality. However, a further quantitative review should consider this limitation and assess the risk of potential biases deriving from selection of participants, data collection and analysis.

## Author contributions

PV took overall responsibility for the conceptualization and design of the review. She revised it critically for important intellectual content. SB and GR searched for the articles in the review, assessed them for relevance. PV, SB, GR, and RT were involved in the interpretation of data, in writing and editing the final article; final approval of the version to be published; agreement to be accountable for all aspects of the work in ensuring that questions related to the accuracy or integrity of any part of the work are appropriately investigated and resolved.

### Conflict of interest statement

The authors declare that the research was conducted in the absence of any commercial or financial relationships that could be construed as a potential conflict of interest.
