# Supplementary material for: Exploring Relationships: A Systematic Review on Intimate Partner Violence and Attachment
Source: Front Psychol. 2018 Jul 5;9:1166. doi: 10.3389/fpsyg.2018.01166 (PMC6042056; doi:10.3389/fpsyg.2018.01166)
Supplement: Supplementary file 1 [file Data_Sheet_1.docx]

**APPENDIX A**

**Final Search:** Concept 1 AND Concept 2

1. **Concept 1: Intimate Partner Violence**

(concept A AND concept B) OR concept C

1. *Intimate relationship*

Spous* OR Intimate OR Dating OR Romantic OR Husband* OR Partner* OR Wife OR Wives OR Marital OR Married OR Pregnan* OR Feminicide OR Domestic OR Conjugal* OR Consort* OR Couple*

1. *Violence*

Abus* OR Aggress* OR Violen* OR Homicide OR Humiliat* OR “controlling behavior” OR “controlling behaviour” OR threat* OR battering OR battered OR offen* OR coertion OR coercitive OR assault* OR maltreat* OR rape* OR beat* OR hurt* OR insult*

1. *Instruments or specific expressions*

IPV OR “gender-based violence” OR “patriarchal terrorism” OR “Coercive Controlling Violence” OR “Violent Resistance” OR “Mutual Violent Control Violence” OR “Separation-Instigated Violence” OR “Male-Controlling Interactive Violence” OR “Conflict Motivated Violence” OR “Episodic male battering” OR “Separation-engendered violence” OR “Conflict Tactics Scales” OR “Abuse Assessment Screen” OR “Violence Against Women Survey” OR “Sexual Experience Survey” OR “Severity of Violence Against Women” OR “Women’s Experience with Battering” OR “Woman Abuse Screening Tool” OR “Composite Abuse Scale” OR “Behavior Risk Factor Surveillance System Module” OR “Norvold Questionnaire” OR “Danger Assessment Scale” OR “Hurt. Insult. Threat. Scream” OR “Psychological Maltreatment of Women Inventory” OR “Humiliation, afraid, rape and kick” OR “Hurt insulted threatened or screamed at questionnaire” OR “Humiliation, afraid, rape and kick” OR “Emotional Abuse Questionnaire” OR “Psychological maltreatment of woman inventory” OR “Psychological maltreatment of partner”

1. **Concept 2: Attachment**

Attach* OR “Experiences in close relationships” OR “Experiences in close relationship” OR “Relationship Style Questionnaire” OR “Relationships Style Questionnaire”
