# Supplementary material for: Exploring Relationships: A Systematic Review on Intimate Partner Violence and Attachment
Source: Front Psychol. 2018 Jul 5;9:1166. doi: 10.3389/fpsyg.2018.01166 (PMC6042056; doi:10.3389/fpsyg.2018.01166)
Supplement: Supplementary file 2 [file Data_Sheet_2.docx]

**APPENDIX B**

**Final Search:** Concept 1 AND Concept 2

1. **Concept 1: Intimate Partner Violence**

(concept A AND concept B) OR concept C

1. *Intimate relationship*

(Spous*[Title/Abstract] OR Intimate[Title/Abstract] OR Dating[Title/Abstract] OR Romantic[Title/Abstract] OR Husband*[Title/Abstract] OR Partner*[Title/Abstract] OR Wife[Title/Abstract] OR Wives[Title/Abstract] OR Marital[Title/Abstract] OR Married[Title/Abstract] OR Pregnan*[Title/Abstract] OR Feminicide[Title/Abstract] OR Domestic[Title/Abstract] OR Conjugal*[Title/Abstract] OR Consort*[Title/Abstract] OR Couple*[Title/Abstract])))

1. *Violence*

(((((Abus*[Title/Abstract] OR Aggress*[Title/Abstract] OR Violen*[Title/Abstract] OR Homicide[Title/Abstract] OR Humiliat*[Title/Abstract] OR "controlling behavior"[Title/Abstract] OR "controlling behaviour"[Title/Abstract] OR threat*[Title/Abstract] OR battering[Title/Abstract] OR battered[Title/Abstract] OR offen*[Title/Abstract] OR coertion[Title/Abstract] OR coercitive[Title/Abstract] OR assault*[Title/Abstract] OR maltreat*[Title/Abstract] OR rape*[Title/Abstract] OR beat*[Title/Abstract] OR hurt*[Title/Abstract] OR insult*[Title/Abstract]))

1. *Instruments, specific expressions and Mesh Terms*

((((("Domestic Violence "[MeSH Terms]) OR "Gender-based Violence"[MeSH Terms]) OR "Intimate Partner Violence"[MeSH Terms])) OR ((IPV[Title/Abstract] OR "gender-based violence"[Title/Abstract] OR "patriarchal terrorism"[Title/Abstract] OR "Coercive Controlling Violence"[Title/Abstract] OR "Violent Resistance"[Title/Abstract] OR "Mutual Violent Control Violence"[Title/Abstract] OR "Separation-Instigated Violence"[Title/Abstract] OR "Male-Controlling Interactive Violence"[Title/Abstract] OR "Conflict Motivated Violence"[Title/Abstract] OR "Episodic male battering"[Title/Abstract] OR "Separation-engendered violence"[Title/Abstract] OR "Conflict Tactics Scales"[Title/Abstract] OR "Abuse Assessment Screen"[Title/Abstract] OR "Violence Against Women Survey"[Title/Abstract] OR "Sexual Experience Survey"[Title/Abstract] OR "Severity of Violence Against Women"[Title/Abstract] OR "Women's Experience with Battering"[Title/Abstract] OR "Woman Abuse Screening Tool"[Title/Abstract] OR "Composite Abuse Scale"[Title/Abstract] OR "Behavior Risk Factor Surveillance System Module"[Title/Abstract] OR "Norvold Questionnaire"[Title/Abstract] OR "Danger Assessment Scale"[Title/Abstract] OR "Hurt. Insult. Threat. Scream"[Title/Abstract] OR "Psychological Maltreatment of Women Inventory"[Title/Abstract] OR "Humiliation, afraid, rape[Title/Abstract] AND kick"[Title/Abstract] OR "Hurt insulted threatened[Title/Abstract] OR screamed at questionnaire"[Title/Abstract] OR "Humiliation, afraid, rape[Title/Abstract] AND kick"[Title/Abstract] OR "Emotional Abuse Questionnaire"[Title/Abstract] OR "Psychological maltreatment of woman inventory"[Title/Abstract] OR "Psychological maltreatment of partner"[Title/Abstract]))))

1. **Concept 2: Attachment**

(((Attach*[Title/Abstract] OR "Experiences in close relationships"[Title/Abstract] OR "Experiences in close relationship"[Title/Abstract] OR "Relationship Style Questionnaire"[Title/Abstract] OR "Relationships Style Questionnaire"[Title/Abstract])))
